# Particle toxicology and health - where are we?

**DOI:** 10.1186/s12989-019-0302-8

**Published:** 2019-04-23

**Authors:** Michael Riediker, Daniele Zink, Wolfgang Kreyling, Günter Oberdörster, Alison Elder, Uschi Graham, Iseult Lynch, Albert Duschl, Gaku Ichihara, Sahoko Ichihara, Takahiro Kobayashi, Naomi Hisanaga, Masakazu Umezawa, Tsun-Jen Cheng, Richard Handy, Mary Gulumian, Sally Tinkle, Flemming Cassee

**Affiliations:** 1 Swiss Centre for Occupational and Environmental Health (SCOEH), Binzhofstrasse 87, CH-8404 Winterthur, Switzerland; 2 Institute of Bioengineering and Nanotechnology, Agency for Science, Technology and Research (A*STAR), Singapore, Singapore; 3 Institute of Epidemiology, Helmholtz Center Munich – German Research Center for Environmental Health, Neuherberg, Munich Germany; 4 Department of Environmental Medicine, University of Rochester, Rochester, NY USA; 5 University of Kentucky, Lexington, KY USA; 6 School of Geography, Earth and Environmental Sciences, University of Birmingham, Birmingham, UK; 7 Department of Biosciences, Allergy Cancer BioNano Research Centre, University of Salzburg, Salzburg, Austria; 8 Tokyo University of Science, Tokyo, Japan; 9 Jichi Medical University, Simotsuke, Japan; 10 National Institute for Environmental Studies, Tsukuba-City, Japan; 11 Aichi Gakusen University, Okazaki, Japan; 12 National Taiwan University, Taipei, Taiwan; 13 School of Biological Sciences, Plymouth University, Plymouth, UK; 14 National Institute for Occupational Health and Haematology and Molecular Medicine, University of the Witwatersrand, Johannesburg, South Africa; 15 Science and Technology Policy Institute, Washington, DC USA; 16 National Institute for Public Health and the Environment (RIVM), Bilthoven, The Netherlands; 17 Institute for Risk Assessment Studies (IRAS), Utrrecht University, Utrecht, The Netherlands

## Abstract

**Background:**

Particles and fibres affect human health as a function of their properties such as chemical composition, size and shape but also depending on complex interactions in an organism that occur at various levels between particle uptake and target organ responses.

While particulate pollution is one of the leading contributors to the global burden of disease, particles are also increasingly used for medical purposes. Over the past decades we have gained considerable experience in how particle properties and particle-bio interactions are linked to human health. This insight is useful for improved risk management in the case of unwanted health effects but also for developing novel medical therapies. The concepts that help us better understand particles’ and fibres’ risks include the fate of particles in the body; exposure, dosimetry and dose-metrics and the 5 Bs: bioavailability, biopersistence, bioprocessing, biomodification and bioclearance of (nano)particles. This includes the role of the biomolecule corona, immunity and systemic responses, non-specific effects in the lungs and other body parts, particle effects and the developing body, and the link from the natural environment to human health. The importance of these different concepts for the human health risk depends not only on the properties of the particles and fibres, but is also strongly influenced by production, use and disposal scenarios.

**Conclusions:**

Lessons learned from the past can prove helpful for the future of the field, notably for understanding novel particles and fibres and for defining appropriate risk management and governance approaches.

## Background

Particles and fibres of various sizes and shapes are important for human health. According to the Global Burden of Disease study [1], in the year 2015, 4.2 million people died as a consequence of ambient particulate matter (PM) exposure, 2.9 million from household air pollution, 0.4 million from occupational PM exposures and 0.2 million from asbestos. However, particles can also have positive health consequences when used for medical purposes such as drug delivery. Over the past decades, an enormous amount of knowledge has been amassed that describes the many different properties of particles and fibres that shape the responses they can evoke in humans and animals.

The growth of the knowledge is well reflected in the series of international meetings on this topic that started with a first conference in the UK in 1979 and has since been held 11 times. The first conferences were dominated by asbestos, crystalline silica and coal dust, which were mostly issues for workers’ health. The focus stayed for a long time in the occupational health domain by looking next at man-made mineral-fibres and asbestos substitutes. The scientific efforts then expanded to include the public health domain with the realisation that a large burden of disease was caused by ambient fine and ultrafine airborne particles. The more recent conferences reflect the change in risk management by no longer just discussing recognised burdens of disease but also anticipating newly emerging risks by discussing widely the potential mechanisms via which engineered nanoparticles could lead to toxic effects.

The most recent conference in Singapore aimed to recapitulate the knowledge we have gained in the field of particle toxicology and to include also the positive aspects of particle-biology interactions that are useful for particle-based medicine. The keynote speakers of the Singapore conference presented the concepts that in combination help understand the large and complex field of particle toxicology and medicine. They helped create the here presented synthesis of the current state of the art and where the field is heading. Discussions will continue at the next conference, which will be in September 2019 in Salzburg, Austria, addressing the theme of “developing solutions for the benefit of workers, consumers, patients and environment”.

## Main Text

### Particle Toxicology

Particle (and fibre) toxicology focusses on understanding and describing the relationship between an exposure to the agents and their capability to adversely affect human health and aims to identify the underlying pathobiological mechanisms. There is a long history of particle toxicology that was recently summarized by Donaldson and Seaton [2]. This substantial body of information on quartz, coal and asbestos has been useful to identify metrics that can be used to predict adverse health effects for new challenges presented, as for example by engineered nanomaterials with structures in the nanoscale (between 1 to 100 nm) and especially nanofibers and nanoparticles (NPs) with two or three dimensions, respectively, in the nanoscale [3–5]. The famous fibre paradigm originates from our understanding on how asbestos can cause lung cancer including mesothelioma. It includes issues like frustrated phagocytosis where the macrophages are not able to fully engulf the fibres, resulting in sustained generation of reactive oxygen species, as well as blocking of the stoma in the chest wall by fibres with subsequent inflammatory responses [6]. The many cases of silica exposure have exemplified that this substance can cause irreversible lung disease known as silicosis. Unlike asbestos, where much effort is put into preventing exposures, silica exposure continues to occur in the 21st century which goes hand in hand with the occurrence of silicosis. The importance of particle solubility was illustrated by silicic acid released from the quartz surface as being the component responsible for the toxicity. At the same time we know that the physical aspects of particles and fibres will have a major role in mechanical irritation in the lungs resulting in inflammation. Although inflammation in itself can be seen as a host response that should protect us, excessive inflammation, especially if it persists over prolonged time, can cause fibrosis, oedema as well as the formation and progression of tumours.

Following the cases of asbestos, coal and silica/quartz, people became very much aware of the health risks associated with being exposed at work to particles and fibres, including man-made mineral and synthetic vitreous fibres. By the end of the previous century (i.e. the late 1990s), scientific attention shifted from occupational exposure to environmental exposures as a number of epidemiological studies indicated that ambient PM exposures were associated with premature mortality and worsening of several diseases. Apart from the known lung diseases like asthma and chronic-obstructive pulmonary disease, cardiovascular diseases and cancer were noted to be impacted by exposure to outdoor PM. The levels of PM at which the associations were reported were well below prevailing air quality standards, and particle toxicology was needed to prove causality and provide plausible biological mechanisms that could explain and support these associations [7]. Although 2-3 decades of toxicological research have taught us a lot, there is still a lot of uncertainty on how low levels of particles can cause so many different effects in humans, and a ranking of substances or sources of emissions can be presented to guide risk management and policy measures is still missing [8].

There are some common features to particle-induced hazard. For example, inhalation of particles can cause oxidative stress which may lead to genotoxicity and reversible or persistent inflammation [9]. Some of the target organs, such as the lungs and the cardiovascular system, are well studied. More recently, it became evident that inhaled particles can also affect the central nervous system [10, 11] and reproduction [12]. This seems to be by and large due to translocation of the smallest particles in the mixture, referred to as ultrafine PM or nanoparticles, into the internal organs [13]. The evidence derived from studies using ultrafine PM has also led to a rapidly growing interest in the toxicology of man-made, manufactured or engineered NPs in the last 15 years. Interestingly, there is a significant overlap in the toxicology of ultrafine particles and engineered NPs and cross talk between these two areas can boost our understanding of the toxicology of nano-sized particles [14].

### Particle Medicine

The field of particle medicine started to emerge about 50 years ago [15, 16]. Research intensified when it became clear that nanoparticles (NPs) have unusual properties with exciting potential for the improvement of established and the development of novel clinical applications. Areas of high interest are imaging and diagnostics, drug delivery and anticancer therapy [17–25]. To date, more than a dozen anticancer nanomedicines have been approved for clinical use, and almost 40 are in clinical trials. Particular attention is currently paid to precision medicine. Here, in case of cancer treatments, the goal is to develop personalized anticancer nanomedicines, which are engineered to target, for instance, a particular type of tumour with a specific location and microvasculature pattern.

Anticancer nanomedicines have various beneficial properties. These include enhanced accumulation in solid tumours with reduced off-target delivery and improved safety. The enhanced permeability and retention (EPR) effect is thought to play a central role in the improved efficacy. The EPR effect is based on leaky blood vessels and impaired lymphatics in tumour tissue, which leads to enhanced extravasation of NPs into tumour tissue and reduced clearance by lymphatic drainage [26–28]. However, the EPR effect would only be relevant with respect to relatively large and well-vascularized tumours, and various challenges remain, including the treatment of leukaemia and metastatic disease. Such challenges must be addressed by more specific targeting strategies. These include surface modifications of NPs with cancer cell targeting ligands, such as folate or antibodies, and magnetic targeting [26, 29–31]. Specific targeting of nanomedicines may also be helpful for addressing the challenge that the EPR was effective in animal models, but it failed, so far, to perform well in human patients [32, 33].

Surface modifications are not only important for targeting, but also for preventing clearance of nanodrugs by phagocytotic cells from the reticuloendothelial system. This is critical for increasing the circulatory half-life of nanomedicines, and for preventing damage of non-target tissues due to activation of resident phagocytotic cells. The classical strategy for reducing interactions with the reticuloendothelial system is to modify the surface of the medical NPs with polyethylene glycol (PEGylation), which hinders opsonisation (binding of proteins recognised by phagocytotic cells), but may shield targeting ligands [34]. Other strategies include biomimetic coating with proteo-lipid membranes extracted from leukocytes [35], platelets [36] or other cells, and autologous cells should be used for clinical applications. Such membranes contain biological functions based on their protein content, which include “marker of self/don’t eat me” signals. A more defined approach is to modify the NP surface with markers of “self/don’t eat me” signals such as the anti-phagocytotic SIRPα ligand CD47 and respective synthetic peptides [37].

Tuning the shape and size of NP medicines is also critical. These features influence the radial drift (margination) of NPs in blood vessels. Whereas small spherical NPs accumulate within the center of blood vessels, disc-like particles display enhanced lateral drift due to tumbling, and also have larger surface areas for endothelial adhesion [38]. Both are important for extravasation in tumour tissue. In addition, NP density appears to influence margination, with enhanced margination of high-density NPs. This leads to an easier distribution of low-density NPs in the body, which is associated with more rapid renal clearance [39].

Renal clearance is also strongly dependent on NP size, shape and charge, not only due to indirect effects based on margination, but also due to the properties of the renal filtration barrier. Rigid spherical NPs are not efficiently cleared by renal filtration if their hydrodynamic diameter exceeds 5.5 nm [40]. Surprisingly, large nanofiber-like materials, such as individualized carbon nanotubes with diameters and lengths of up to 20 – 30 nm and 500 – 2000 nm, respectively, easily cross through the renal filtration barrier when aligned in the right orientation, and are cleared with similar efficiency as small molecules [41, 42]. Tuning of renal clearance of nanomedicines must be carefully adjusted to keep the balance between maintaining therapeutic plasma levels and safe elimination from the body.

During the last decades, nanomedicines with a wide range of structural features have been developed for the delivery of small molecule drugs, biologics, nucleic acids, or co-delivery of multiple compounds [24, 43]. The first approved nanomedicines for drug delivery were a synthetic polymer conjugated to the anticancer protein neocarcinostatin [44], and PEGylated liposomal doxorubicin (Doxil/Caelyx) [45]. Since the 2000s, polymeric micelle-based nanomedicines for drug delivery have been approved for clinical applications. Polymeric micelles consist of amphiphilic block copolymers that self-assemble into a core-shell structure. The hydrophobic core can be loaded with hydrophobic small molecule drugs or biologics, whereas the hydrophilic shell can be further modified by PEGylation or with targeting ligands.

Until now, polymeric micelles or other types of NPs, which did not have any anticancer activity themselves, were used as vehicles for the delivery of anticancer agents. An exciting more recent development are micellar nanocomplexes (MNCs) for anticancer protein drug delivery, which have anticancer activity themselves (also in the absence of the anticancer protein) [46]. The MNCs were based on derivatives of the green tea catechin (-)-epigallocatechin-3-O-gallate, which has known anticancer activity. After loading of the MNCs with the anticancer protein drug Herceptin, synergistic anticancer activity of the MNCs and Herceptin has been observed, which resulted in enhanced antitumor activity *in vitro* and *in vivo* compared to Herceptin alone [46]. In addition, prolonged plasma half-life and enhanced accumulation in the tumour tissue of the MNCs compared to the protein drug alone were demonstrated [46].

The most important goal for the future is to achieve personalized treatments by designing precision nanomedicines. Crucial for achieving this goal is an improved understanding of the interactions between NPs and biological structures and tissues. This will help to guide the development of smart strategies for manipulating NP surface chemistries, which is required for reducing unspecific interactions and for increasing targeting of tumors with specific, individual properties. Replacement of conventional synthetic polymers by bio-inspired molecules, such as antibodies for targeting and “don’t eat me” signals, will play a central role in developing smarter and personlized strategies. Important will be also the tuning of the size and shape of nanomedicines based on the understanding of how these features influence interactions in normal and disease states., Smarter strategies for tuning size and shape will include further development of multistage and tumor environment-responsive NPs, with larger parental NPs that have improved circulatory half-life, which release smaller NPs at the tumor that can easier penetrate into the tumor tissue [47, 48]. Addressing the variability between patients as a function of age, gender, ethnicity, their individual disease state and other patient-specific factors will be essential to guide the design of NPs for precision nanomedicine and to achieve personalized treatments.

### Dynamic Fate of Inhaled Nanoparticles in the Lungs of Rodents

**Fig. 1:**
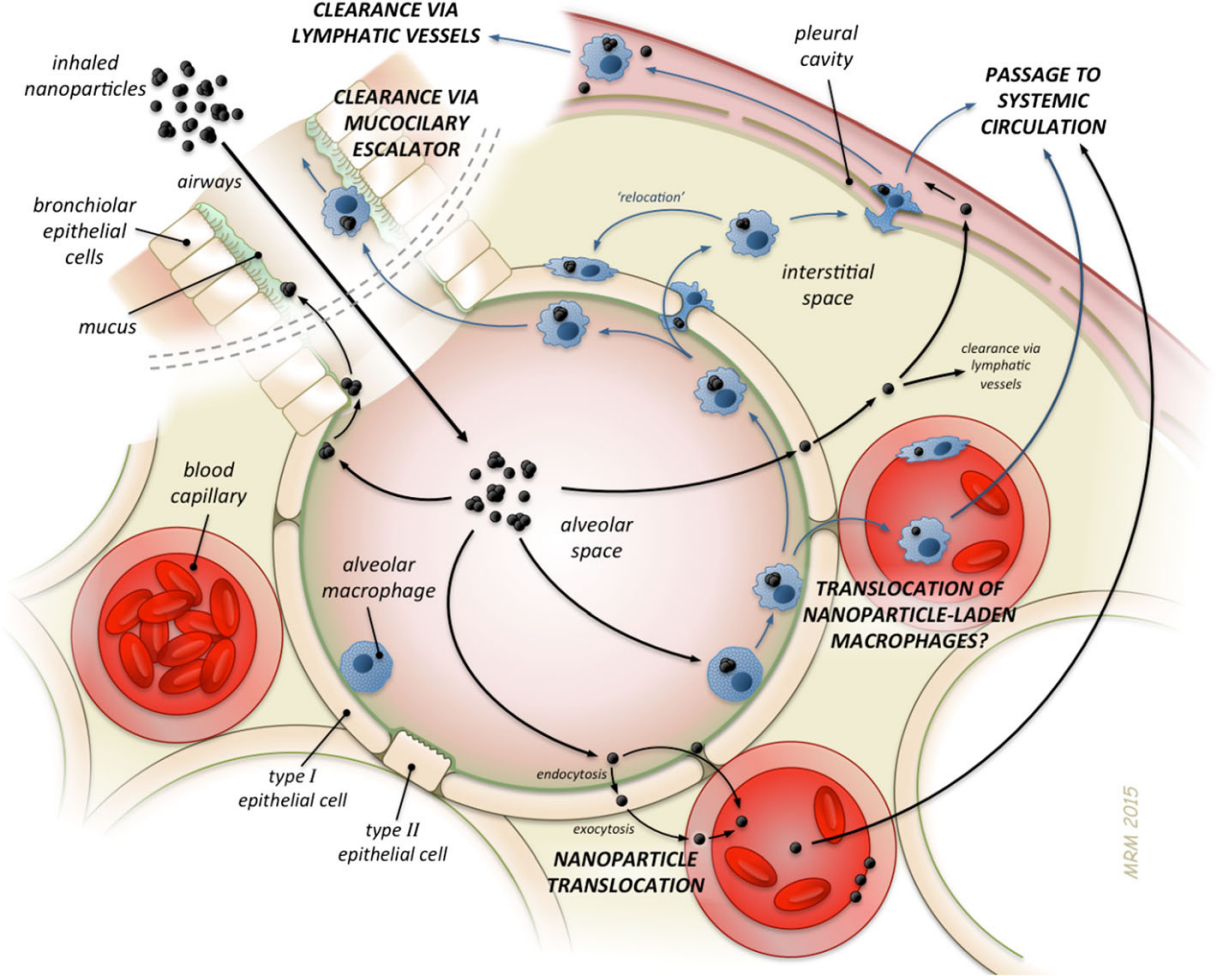
Overview on different types of NP’s translocation and clearance in the lungs. Artwork by Mark Miller, reproduced with permission from [14].

Deposition of inhaled NPs is governed by their diffusivity in the air leading to a rather homogeneous deposition density on the epithelium of the various regions of the respiratory tract. As a result, the deposited fraction in the lungs reaches a maximum of about 50% of the inhaled aerosol at a size of 20 nm [49, 50] with about 15% alveolar deposition and 35% depositing in the conducting airways of the head and thorax. In other words, two thirds of the deposited 20 nm sized NP will be cleared rapidly within 24 hours by mucociliary action within the ciliated conducting airways and one third will be long-term retained in the lung periphery. Below that size, increasing fractions deposit in the airways of the head and thorax according to their increasing diffusivity with decreasing size, such that less NP reach the distal alveolar region (see Fig. 1). In this short summary a few consequences on the biokinetics fate will be discussed for insoluble NP.

#### Relocation and re-entrainment of Inhaled NPs in the rodent lungs

**Fig. 2:**
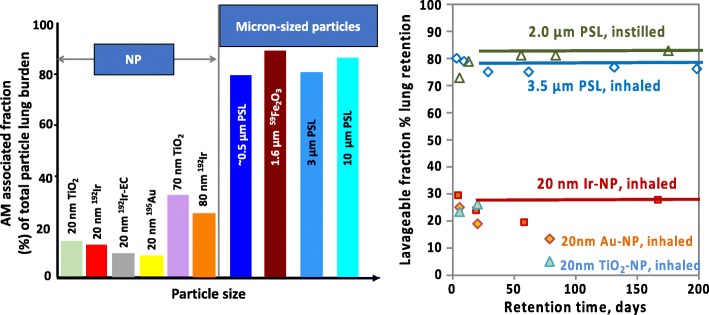
Panel A: Alveolar-macrophage (AM) associated percentages of inhaled NP (20 + 80 nm iridium NP, 20 nm gold + elemental-carbon NP and 20 + 70 nm titanium dioxide NP) versus instilled micron-sized particles (0.5, 3, and 10 μm polystyrene (PSL) particles) found in bronchoalveolar lavage (BAL) of rats 24 h after application [54]. Panel B: Percentages of inhaled NP (20 nm iridium NP from 3 - 180 days and 20 nm gold + titanium dioxide NP from 3 - 28 days after inhalation) found in bronchoalveolar lavage fluids of rats at various time points [54] versus micron-sized particles (either inhaled 3.5 μm PSL [52] or intratracheally instilled fluorescent 2 μm PSL [51]. All percentages are relative to the contemporary lung burden.

In rodents, there is evidence that rather constantly about 80% of micron-sized particles can be lavaged from the lung surface during six months post-exposure (p.e.) when the lavaged particle fractions are normalized to the contemporary lung burden at each time point [51–53]. In contrast, only 20% of 20-nm-sized NPs and about 30% of 70-nm-sized NP deposited during inhalation or instillation can be lavaged – see Fig. 2 [54–59].

The low NP recovery becomes plausible since NPs deposit rather uniform on the surface of an alveolus by diffusion. Indeed, macrophages on the epithelial surface will rapidly phagocytize all NP which happen to be deposited close to where a macrophage happens to reside, while distant NP are not recognized due to the weak opsonizing signal of NPs. However, since only about 10 – 100 macrophages are residing in an alveolus of 300 nm diameter of a healthy rodent [60], these surface macrophages cover only a fraction of <0.001 of the alveolar surface [57]. Since epithelial Type I+II cells possess well-identified endocytotic mechanisms of NP uptake and less so for micron-sized particles their endocytic NP uptake capacity competes with the phagocytic capacity of surface macrophages. As discussed in our previous paper [59], numerous authors provided evidence that epithelial type 1 cells (EC1) can endocytose NP at the epithelial surface and eventually exocytose those towards the baso-lateral side at the end of the last century, including the review of Lehnert [61] and papers by Adamson and Bowden [62–64]. More recently, Thorley and co-workers [65] provided evidence for prominent NP uptake by EC1 while they ruled out uptake by alveolar epithelial type 2 cells (EC2) and the passage of NPs by paracellular transport was also discounted. They stated that NP uptake occurs preferentially by diffusive processes into the cytoplasm which allows for exocytosis and transport across the basal membrane into the interstitium, as supported by *in vivo* studies. In collaborative studies [66, 67], localization of AuNP and TiO_2_-NP in interstitial spaces was demonstrated by Transmission Electron Microscopy (TEM). According to [68, 69] the cytoplasmic leaflets of EC1 provide the largest portion of their surface area on the basal membrane which separates the adjacent vascular endothelial cells to allow unhindered gas exchange. Yet, data up to 24 hours do not support the notion that neither AuNP nor TiO_2_-NP cross EC 1 at both - the “active site of gas exchange” [68] and the basal membrane, since any NP exocytosis would lead to rather rapid uptake by vascular endothelial cells and translocation into the circulation which was not observed. Instead the translocated AuNP or TiO_2_-NP fractions to blood were rather small during the first 24 h p.e.. Hence, EC1 either exocytose directly into septal interstitial spaces – which provide only a relatively small surface area at the side of EC1 – and/or the exocytosed NP migrate in between the basal membrane and the EC1membranes to the next septal interstitial space. Once there, NP may be phagocytized by interstitial macrophages (IM), fibroblasts, etc. Referring to [61, 70] there is a large population of IM such that the role of IM in phagocytizing NPs and long-term retention in the septal interstitial spaces appears plausible. For gradual re-entrainment back onto the epithelium, Lehnert [61] reviewed the pathway of IM onto the epithelium. In addition, IM and fibroblasts may exchange their NP-load with AM like in a relay. Hence, NP behave differently compared to micron-sized particles which are retained on the surface of the rodent epithelium to be eliminated by long-term, macrophage-mediated clearance (LT-MC) at a rate of initially 2-3%/d which declines gradually over time [54, 71, 72]. Surprisingly, NPs are cleared with a similar clearance rate dynamics via this macrophage-mediated transport indicating re-entrainment of the NPs from the interstitial spaces back on top of the epithelium for subsequent LT-MC towards the larynx and into the gastro-intestinal-tract (GIT) [55, 56]. Re-entrainment may occur across the alveolar epithelium and/or via interstitial-lymphatic clearance to bronchus-associated lymphoid tissue (BALT) entering the epithelial surface at the airway epithelium for clearance by surface macrophages, as hypothesized several decades ago [52, 73–76]. NP passage through BALT at bronchioles-alveolar duct junctions back onto the bronchiolar epithelium [63] [77] cannot be excluded; however, BALT may play an important immunogenic role for fluid absorbed from the alveolar surface, but the reverse flow onto the epithelial surface was postulated in the literature but has not been proven so far [68, 69]. Furthermore, there are only about 30-50 BALT sites in the rat lungs [61, 78] which are far too few compared to the number required for NP re-entrainment and LT-MC clearance.

As a result, LT-MC is the most prominent long-term clearance mechanism for insoluble NPs from the peripheral lung of rodents. In fact, although the NP are retained in the septal interstitial spaces close to blood vessels, only rather small fractions are translocated via this pathway into circulation leading to subsequent accumulation in secondary organs and tissues which, however, depends strongly on the physicochemical properties of the NPs. For example, four different materials (iridium (Ir), elemental carbon (EC), TiO_2_, and gold (Au)), which were inhaled as freshly generated 20-nm NP aerosols during a single 1–2 h exposure by healthy, adult, female Wistar–Kyoto rats; the translocation percentages (normalized to the alveolar NP deposition) of IrNP (7.96 ± 0.47) and TiO_2_NP (6.95 ± 0.14) were significantly higher than those of elemental carbon (2.18 ± 0.31) and AuNP (1.79 ± 0.39) as shown in Table 12.1 of [79].

Retention in secondary organs was followed up to six months after the inhalation of the IrNP aerosol showing no detectable clearance [55, 58]. During chronic exposure to insoluble NP, continuous accumulation is likely to occur in secondary organs. This may play a modulating role in adverse cardio-vascular health effects which have been observed in epidemiologic studies after exposure to ambient fine and ultrafine particles [80].

#### NP pathways from lungs to circulation and accumulation in secondary organs and tissues

Systemically circulating NPs may accumulate in secondary organs and tissues by two particle clearance pathways, (i) NP translocation across the air-blood-barrier (ABB) either directly into blood circulation or via the thoracic lymph duct and (ii) NP absorption across the GIT walls, again either directly into blood circulation or via the thoracic lymph duct, of those NP which were eliminated from the lungs towards the larynx and swallowed into the GIT. The latter clearance pathway has a fast component of those NP deposited on the epithelium of the conducting airways which are cleared rapidly by mucociliary action (MCC) followed by a slow component of those NP from the peripheral lungs eliminated by LT-MC towards the larynx.

**Fig. 3:**
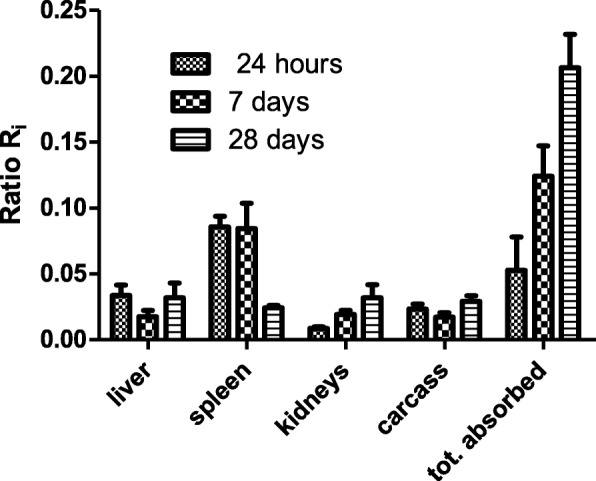
The ratios R_i_ represent the fractions of TiO_2_2NP present in liver, spleen, kidneys and carcass (without organs) and the integral sum of all absorbed fractions determined after IT-instillation that have been absorbed through the GIT relative to the sum of gut-absorbed and ABB-translocated TiO_2_NP after 1, 7 and 28 days. Mean ± SEM of n=4 rats at each time point.

The contribution of both pathways towards secondary organs was quantitatively investigated for the first time in a series of studies in which identical 70-nm-sized TiO_2_NP were applied to rats either as a single bolus via intratracheal (IT) instillation or via gavage (oral ingestion) or via intravenous injection. Their biokinetics were determined quantitatively in the entire organism during the next 28 days [81–83]. The biokinetics data obtained from the gavage study were used to estimate the absorbed TiO_2_NP fractions across the gut walls after IT-instillation which had been cleared from the lungs via the larynx into the GIT. In Fig. 3 the ratios Ri of gut-absorbed and subsequently accumulated TiO_2_NP divided by the sum of both – gut-absorbed and ABB-translocated TiO_2_NP - are shown for liver, spleen, kidneys and the carcass (comprising of skeleton, muscles, fat, skin, but without organs) and the integral absorbed TiO_2_NP fraction at different time points between 1 and 28 days after IT-instillation. The integral absorbed TiO_2_NP ratios increase with time up to 0.2 of all systemically circulating TiO_2_NP due to the continuous LT-MC transport leading to continuous absorption across the gut walls. Ratios in liver, kidneys and the various tissues of the carcass stay below 0.05, but the absorbed TiO_2_NP ratios in the spleen are about 10.1 at days 1 and 7. These data show that accumulation in secondary organs and tissues is predominantly determined by ABB-translocated TiO_2_NP which, however, occurs mainly during the first few days after IT-instillation (see Fig. 3). Yet, with increasing retention time the gut-absorbed NP fractions become more and more important.

### Interrelated Concepts of Exposure, Dosimetry and Dose-Metrics for NP Risk Assessment

Results of numerous studies, *in vitro* and *in vivo*, have revealed that engineered NPs and ambient ultrafine particles (UFPs, e.g. diesel exhaust) can induce significant dose-dependent toxicity in primary and secondary organs. In order to characterize appropriately the associated risk as a function of hazard and exposure, exposure-dose-response relationships have to be established. With respect to inhalation as the main route of exposure - involving acute, subchronic or chronic rodent studies - a minimum of three exposure concentrations plus sham-exposed controls should be used [84]. The experimental design should include detailed aerosol characterization and biokinetic data. Essential for the evaluation of the results of inhalation studies is to determine the retained dose (lung burden) at the end of exposure to establish dose-response relationships. This is a fundamental requirement which is often forgotten when reporting results only as exposure-response data. Expressing the dose by different metrics such as particle mass, surface area, volume, or number, will provide additional information about potential underlying toxicological mechanisms that control outcomes. The revised OECD Test Guideline 413 [85] for subchronic rodent inhalation now includes a requirement to determine retained lung burdens.

Short-term inhalation studies (STIS, i.e., 5 days) are useful for hazard identification and ranking. Full risk characterization, however, requires subchronic (90-day) or chronic (up to 2 years) studies. Because of the associated ethical concerns (requiring large number of animals) and high costs of longer duration exposures, subacute (28-day) studies with sufficient post-exposure recovery time have been suggested as replacements for longer-term studies. Their usefulness for risk characterization still needs to be validated however [84]. Table 1 lists objectives and design for mammal (predominantly rodent) inhalation studies of different duration.

**Table 1:** Objectives and design for rodent inhalation studies of different duration, modified from [84]

Acute /Subacute 5-28 days)	Subchronic (90 days)	Chronic (2 years)
• To obtain hazard ID and ranking (ideally compared to positive and negative controls) • May be preceded by i.t. instillation or 1 day inhalation with range of doses to estimate inhaled concentration with MPPD model • Ensure rodent-respirable aerosol stability over a range of concentrations • If available use workplace or consumer exposure data to inform aerosol generation • To determine concentrations for 90-day exposures (range-finding) • To collect biokinetic data for portal of entry, and possibly identification of secondary target organs, incl. pleura, and fetus • To provide guidance of dose levels for mechanistic in vitro testing, incl. secondary organs • Post-exposure observation period desirable (~2 months)	• To derive NOAEL • Use minimum 3 concentrations, including known or expected human exposure levels; both sexes optional • If no effect at 50 mg/m^3^ rodent respirable aerosol, then no need to do chronic study • To identify hazard: total respiratory tract, pleura, cardiovascular, central nervous system (CNS), bone marrow • To identify target organs • To select concentration for chronic study • Detailed biokinetics: retention, clearance, organ accumulation, • To predict long-term effects • To inform human risk assessment via dosimetric extrapolation • Post-exposure observation period to assess progression-regression (~3 months)	• To determine long latency effects (cancer); life shortening; extrapulmonary target organs • 3 concentrations based on 90-day or range-finding study results; include human exposure level; high dose: MTD; low dose: no significant effect • To assess total respiratory tract, pleura and systematic effects, nose to alveoli, cardiovascular, CNS, bone marrow, others (reproductive?) • To determine detailed biokinetics: respiratory tract retention, clearance, organ accumulation • To perform extrapolation to human for risk assessment • Post exposure observation period up to a total study duration of 30 months (if survival of ≥20%)

Establishing toxicologically well-characterized particles as positive and negative “Benchmark Materials” [86] for a comparative Hazard and Risk Characterization will facilitate the grouping of inhaled particulate materials which are tested in subacute, subchronic or chronic inhalation studies. This involves comparing the slope of dose-response curves (potency) or the No Observed Adverse Effect Levels (NOAELs) of rodent studies to obtain a hazard ranking. If only a Lowest Observed Adverse Effect Level (LOAEL) can be identified in the rodent study, benchmark dose (BMD) analyses are appropriate. Expressing the retained lung burden by different dose-metrics (particle mass; surface area; number) will help to identify the most appropriate metric by comparison to toxicologically well-characterized benchmark materials. For example, if the measured response to different particle sizes of the benchmark material shows the same correlation with a chosen dose metric, then this metric has better predictive value than other dose metrics. The more predictive metric, then, should be used for evaluating the unknown nanomaterial in comparison to the benchmark material. For poorly soluble particles, surface area has been found to be of best predictive value [87, 88].

As a first step of risk assessment using results of a rodent study, the exposure–dose–response relationship can be analyzed by using a BMD approach in order to derive an associated benchmark concentration (BMC) as a “safe” exposure level for rodents [89]. Results derived from a rodent study can then be the basis for risk extrapolation to human exposure scenarios by deriving a Human Equivalent Concentration (HEC), provided that species differences in respiratory tract dosimetry and retention kinetics are considered [90]. The HEC is defined as the exposure concentration predicted by modelling to result in the same normalized retained lung burden as measured in rodents after acute, subchronic or chronic inhalation. Normalization of deposited or retained dose is frequently done using species-specific lung weight or alveolar surface area. Effects, however, may be different between the species due to different sensitivities or mechanisms of uptake and effect. To account for this and possible toxicokinetic/toxicodynamic differences, assessment or uncertainty factors may have to be included [91].

**Fig. 4:**
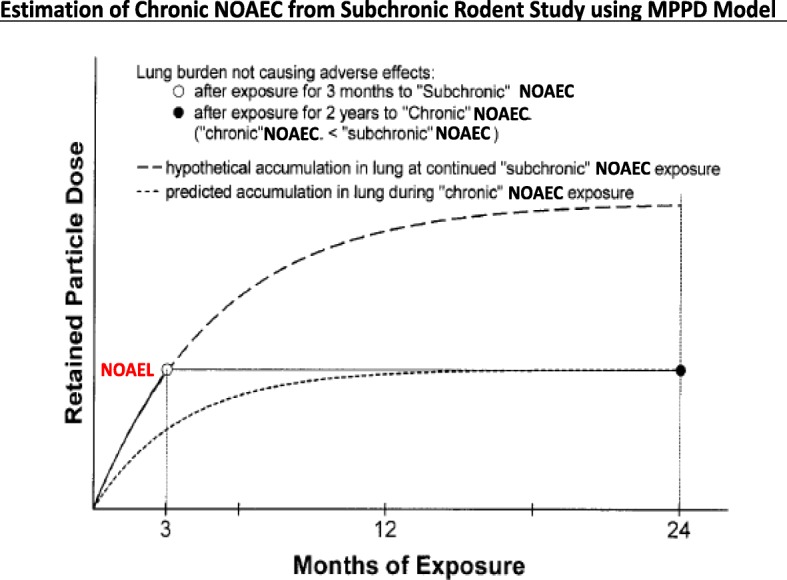
Estimation of chronic NOAEC from subchronic rodent study using the MPPD Model.

Dosimetric extrapolation of results from rodent inhalation studies for deriving the HEC is achieved with the MPPD (Multiple Path Particle Dosimetry) Model [92]. Important recent refinements of MPPD include improved input values for allometrically adjusted respiratory parameters [93] and the choice of specific rat and mouse strains. A suggestion of how to extrapolate a NOAEL and associated exposure concentration (NOAEC) determined in a subchronic rodent inhalation study to a chronic 2-year study has been proposed by [94, 95]. The approach is shown in Fig. 4, it involves the use of the MPPD model to estimate a NOAEC which, after two years of exposure, results in the same NOAEL that was determined in the subchronic study. Such dosimetric extrapolation will avoid the use of an uncertainty factor for extrapolation from subchronic exposure to chronic exposure.

The deposition of airborne particles is affected by the effective or actual density of aerosols, which makes it an important input variable for the MPPD model. Specifically, if the aerosol consists of agglomerated and aggregated NPs, the void spaces between the individual particles of an aerosol cluster change the effective aerosol density to be significantly less than the material density. Whereas a number of methods have described how to measure effective aerosol density (e.g., [96–99], a simple approach for measuring the actual density present in a rodent inhalation study is to perform a short-term inhalation, sacrifice the animals at the end of exposure and measure the deposited lung burden. This allows calculation of the deposition fraction. One then runs the MPPD model with rat specific and body weight allometrically adjusted inputs and changes the input value for the density iteratively until it fits the calculated deposition fraction. Work on this approach is ongoing.

Among the intrinsic physico-chemical properties that impact on NP toxicity, surface properties are, in theory, most influential because of the direct interaction of the particle surface with cells and subcellular components. In addition, extrinsic or functional NP properties, such as specific surface reactivity and dissolution rate are important for categorization and grouping of NPs. With respect to dissolution, NPs are often grouped by their solubility in water [100]. However, water solubility is not always appropriate for predicting *in vivo* dissolution. Obviously, dissolution rates of NPs can vary widely, which has to be considered because the biopersistence will be affected depending on the dissolution rate. There are two basic approaches to determine solubility/dissolution of particles in cell-free systems: the static system, determining the equilibrium solubility, and the dynamic system, determining dissolution rates. The latter mimics more closely the *in vivo* situation, whereas the former can reflect *in vitro* conditions. The composition of the dissolution fluid is another critical factor with respect to closely simulating *in vivo* conditions, e.g., phagolysosomal fluid (pH ~4.5) or epithelial lung lining fluid (pH ~7.4). The significance of reliably assessing the *in vivo* dissolution rate of NPs lies in the possibility to characterize their biopersistence by estimating the overall pulmonary clearance rate and retention halftime. This will be necessary for subsequent use as inputs into the MPPD model for purposes of human risk extrapolation modelling.

**Fig. 5:**
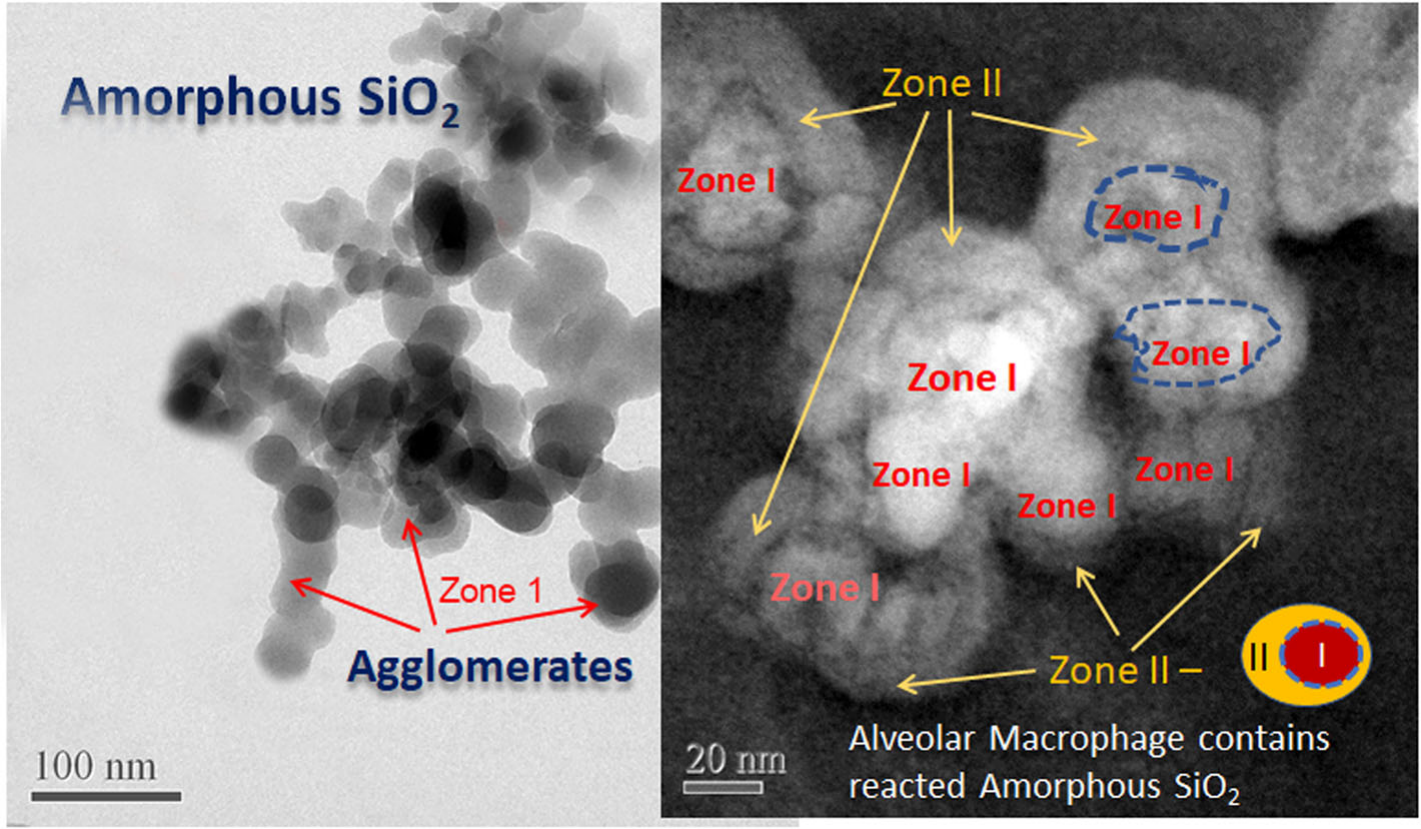
Bioprocessing of inhaled nano-SiO_2_ particles: (left) large agglomerates of amorphous precursor material; right) dark field STEM image showing breakdown of SiO_2_ NPs in alveolar macrophage (Zone 1) and formation of Zone II.

Furthermore, indications from acellular assays that dissolution in the respiratory tract takes place raises the question about the fate and underlying mechanisms by which tissues respond to the dissolving NPs. Bioprocessing or biotransformation events investigated using High Resolution Transmission Electron Microscopy (HRTEM), Scanning transmission electron microscopy (STEM) and Electron energy-loss spectroscopy (EELS) analyses revealed subcellular NP dissolution and chemical interactions resulting in secondary very small NPs [101]. Amorphous nano-silica (SiO_2_) in alveolar macrophages examined by HRTEM after subchronic inhalation in rats at 27 days post-exposure period had undergone significant *in vivo* breakdown and transformation [102]. In particular, a portion of the original NPs were partially dissolved and secondary SiO_2_-reaction zones (precipitates) formed as a result of *in vivo* processing (also called bioprocessing – see section “The 5Bs” below) as shown in Fig. 5 ([102], previously not published). Compared to the starting materials, the bioprocessed SiO_2_ particles showed dissolution patterns (voids/pore formation) and outward growth of reaction zones. The degree of *in vivo* processing of NP can be evaluated with HRTEM which, coupled with dose-response monitoring, could provide further information for NP risk assessment.

As a look to the future, exploration of the physicochemical changes at the particle surface over time following exposure, i.e., bioprocessing, will improve our understanding of tissue dosimetry, biodistribution, and, ultimately, the mechanisms by which inhaled particulates exert toxicological effects.

### The 5 Bs: Bioavailability, biopersistence, bioprocessing, biomodification, and bioclearance of nanoparticles and the role of the biomolecule corona

For drugs and molecular chemicals, the proportion of a drug or other substance which enters the circulation when introduced into the body, and so is able to have an active effect, is considered the bioavailable dose, while the length of time that a molecule (typically a toxicant) remains in the biological organism without being metabolised or excreted is termed biopersistence. Clearly, the route of introduction will affect this proportion, with direct intravenous injection resulting in close to 100% bioavailability, while oral or nasal introduction for example, might result in lower bioavailability due to the biological barriers that exclude some of the compound. Given some of the unique aspects of NPs, however, such as their tendency to agglomerate at higher concentrations [103], their tendency to interact with biomolecular constituents of their surroundings to form so-called biomolecular coronas [104–106], and the size-modulated cellular uptake mechanisms [107, 108], the bioavailable dose for NPs is poorly understood compared to that for molecular substances, leading to enormous challenges in determining meaningful dose-response curves [109, 110]. Additionally, the dynamic nature of NPs and their tendency to evolve and transform during storage [111] or upon interaction with media [112] makes dose characterisation challenging. Indeed, due to interactions with their surroundings, the dispersion media play an important role in defining the NPs dose, but also in determining the NPs “biological” identity, which is the set of proteins and other biomolecules associated with the NP and specifically the outer surface of the absorbed corona which forms the interface to engage with biological receptors [113–116]. Indeed, proteins such as Bovine Serum Albumin (BSA) have long been used as agents for dispersal of NPs for toxicity assessment [117], as has its environmental equivalent, natural organic matter (NOM) for ecotoxicological studies [118, 119]. However, in some cases, adsorption of biomolecules induces partial agglomeration of the NPs thus altering the bioavailable dose [120]. The absorbed biomolecule corona can also alter the biopersistence of the NPs, for example, by changing the rate of dissolution of NPs. For example, the corona adsorbed to NPs has been shown to slow dissolution by blocking oxidative processes [121] or by promoting sulfidation process and entrapping nanocrystals of Ag_2_S in the hard corona [122], while in other cases, adsorbed coronas have been found to accelerate dissolution, especially where there is a strong affinity for the metal ions by the proteins and an excess of the proteins [123].

Recent work using the model organism *Daphnia magna* has shown that proteins secreted by the *Daphnia* into the media induce some agglomeration of NPs but since this brings the NP-agglomerates into the size range of *Daphnia*’s normal food source, it appears to increase uptake / bioavailability of the NPs [108]. Similar findings have suggested that cellular response to the presence of NPs also involves secretion of proteins in response to the initial form of the NP taken up by the organism, causing the “initial” corona to evolve, which can lead to altered uptake and impacts not currently considered in assessing toxicity [124]. Thus, NPs interacting with living organisms are dynamic systems where the NPs affect the organism and the organism affects the NPs. This suggests that there is more than one biological identity for each NP and that this identity evolves as the NPs interact and are internalised and processed by organisms and cells. Indeed, early work to model the corona evolution experimentally, whereby NPs were sequentially incubated in biological fluids representing the external environment (serum) and the cellular environment (e.g. cytosolic fluid) indicated that the resulting corona had proteins from both fluids [125]. .

The physicochemical changes that particles undergo in tissues following exposure, which include dissolution, deagglomeration, secondary particle formation, etc., have been termed bioprocessing. As noted above, the highly reactive surface area of NPs leads to adsorption of a biomolecule corona, which plays a key role in the subsequent bioprocessing of NPs, determining for example the rate of dissolution, the uptake mechanism, and the subsequent biodistribution. From the discussion of the biomolecule corona above, it is clear that the decision as to which bioprocessing step occurs is determined by the biomolecule signals that are associated with the NPs. For example, it has long being recognised that certain proteins trigger recognition by phagocytic cells, leading to rapid clearance of NPs associated with these proteins, which are known as opsonins [126]. Examples of proteins known to enhance phagocytotic uptake include collectin molecules such as surfactant protein A (SP-A) and SP-D, as well as members of the complement cascade involved in wound healing. On the other hand, enormous effort in nanomedicine has been devoted to the development of so-called stealth NPs that can evade the immune system and avoid uptake by phagocytes, either by reducing overall protein binding or by selectively binding the so-called deopsonising proteins such as albumin [34]. Clearly, the specific proteins that bind to NPs will be influenced by the route of internalisation, with the lung surfactant proteins being the main candidates for binding following inhalation, serum proteins being the first binders in the case of intravenous exposure, and a range of enzymes and food biomolecules being potential corona constituents for gastric exposure [127]. Other transformations included under the broad term bioprocessisng include enzymatic digestion [128], such as has been reported for carbon nanotubes [129] and graphene materials [130].

Studies of the biodistribution of NPs as a function of exposure route correlate with this assumption that biomodification, as determined by the NP-associated biomolecules, strongly influences the distribution of the NPs. For example, *in vivo* studies using radiolabelled gold NPs in rats indicated that different exposure routes led to different biodistributions of the NPs, which is most likely a result of different biomolecule adsorption and thus different bioprocessing signals [131, 132]. Also, radiolabelled Au NPs in different sizes (1.4-200 nm) exposed by intra-oesophageal instillation into healthy adult female rats resulted in detectable NPs (ng/g organ) in the stomach, small intestine, liver, spleen, kidney, heart, lung, blood and brain after 24 h as measured by gamma-spectroscopy, with the highest accumulation in secondary organs being for the smallest particles, while the 18 nm particles showed a higher accumulation in brain and heart compared to other sized particles [132]. On the other hand, Au NPs delivered tracheally to rats resulted in the majority of NPs remaining in the lungs (> 95% of the initial dose, ID) with < 1% of the ID translocated to the kidneys, liver, blood and urine, and < 0.01 of the ID reaching the spleen, uterus and heart [131]. While these studies did not explicitly attempt a comparison on the basis of adsorbed biomolecules, it is clear that such a study, including recovery of the NPs and assessment of their biomolecule coronas following translocation and final localisation, would shed important new light on the biomodification of NPs and the role of the biomolecule corona in the bioprocessing steps. Indeed, the hope for both nanomedicine and nanosafety is to “design” NP surfaces to acquire the desired corona to direct the bioprocessing to minimise the risk of harm to humans. Designing the NP surface to tailor the corona is already underway, via the design of stealth particles as discussed above, or indeed via surface modification with small molecules that induce protein-misfolding in a component of the NP-associated protein corona, and which enhances or reduces the NPs’ susceptibility to cell-specific receptor-mediated endocytosis [133]. Such unfolding could lead to unintended immune responses though via display of so-called cryptic epitopes [134], and thus such approaches needed to be conducted with care.

The term biomodification is normally used to indicate some modification of a biological organism, e.g. via genetic or mechanical means, while we use the term to understand the impact of the NPs on the biological organism‘s biochemistry. Thus, we attempt to distinguish between physicochemical transformations of the NP that occur post uptake (biotransformation, bioprocessing) and cellular processes that result in the incorporation of the NP degradation products into existing biological pathways.[128–130] Biomodification pathways, via which the degradation products can be incorporated into existing biological pathways, are especially interesting in terms of the design of safer NPs. One example of a biomodification pathway, proposed for iron oxide NPs, showed that 10 nm iron oxide NPs were degraded in macrophages and the resulting free iron was transformed to ferritin and hemosiderin iron-protein complexes and used to make haemoglobin and myoglobin [135]. In this case the biotransformation also leads to bioclearance of the NPs. Other biomodification routes include lysosomal degradation of NPs as a result of the low pH in the lysosomes coupled with their high enzymatic composition and indeed their role as the “dustbin” of the cell.

The composition of NPs can be conceptually divided into the often inorganic core; the engineered surface coating comprising of the ligand shell and optionally also bio-conjugates; and the corona of adsorbed biological molecules [136]. Empirical evidence shows that all three components of NPs (core, shell and corona) may degrade individually *in vivo* and can drastically modify the life cycle and biodistribution of the whole heterostructure, such that the biodistribution and fate of each sub-component would need to be analyzed individually for regulatory and nanomedical approval purposes [136]. Approaches to do that, based on stable isotope and radiolabelling of core and shell separately are emerging, with differential biomodification process demsonstrated for polymer-coated FeO_x_ NPs [136] versus Au NPs [137]. Thus, approaches to assess biomodification, bioprocessing, and bioclearance are emerging, and these are intrinsically linked. The co-evaluation of core and shell need to be determined on a case-by-case basis until predictive models can be developed.

A range of studies have looked at the correlation between NP properties such as size and surface charge with uptake, biodistribution and bioclearance (defined here as removal from specific organs (e.g. the lung or gut) or from the organism overall. Blanco, Shen and Ferrari looked at effect of size (<5nm, 20-150nm and > 150nm), shape (20-150nm spheres, rods and discs) and surface charge (20-150nm spheres with negative, neutral or positive) on where the NPs accumulated [138]. The findings indicated that the kidney was the main target organ for <5nm NPs, whereas for the 20-150nm spheres, positive charge correlated with enhanced accumulation in liver and spleen. Discs appeared to show most accumulation, collecting in liver, spleen and lungs, with the order of accumulation for shapes being discs > rods > spheres [138]. There is clear evidence that each of these parameters also influences the nature of the biomolecules bound [139–141], and that different coronas lead to different biodistributions of NPs *in vivo*, as indicated above and demonstrated by Wang et al. [142]

**Fig. 6:**
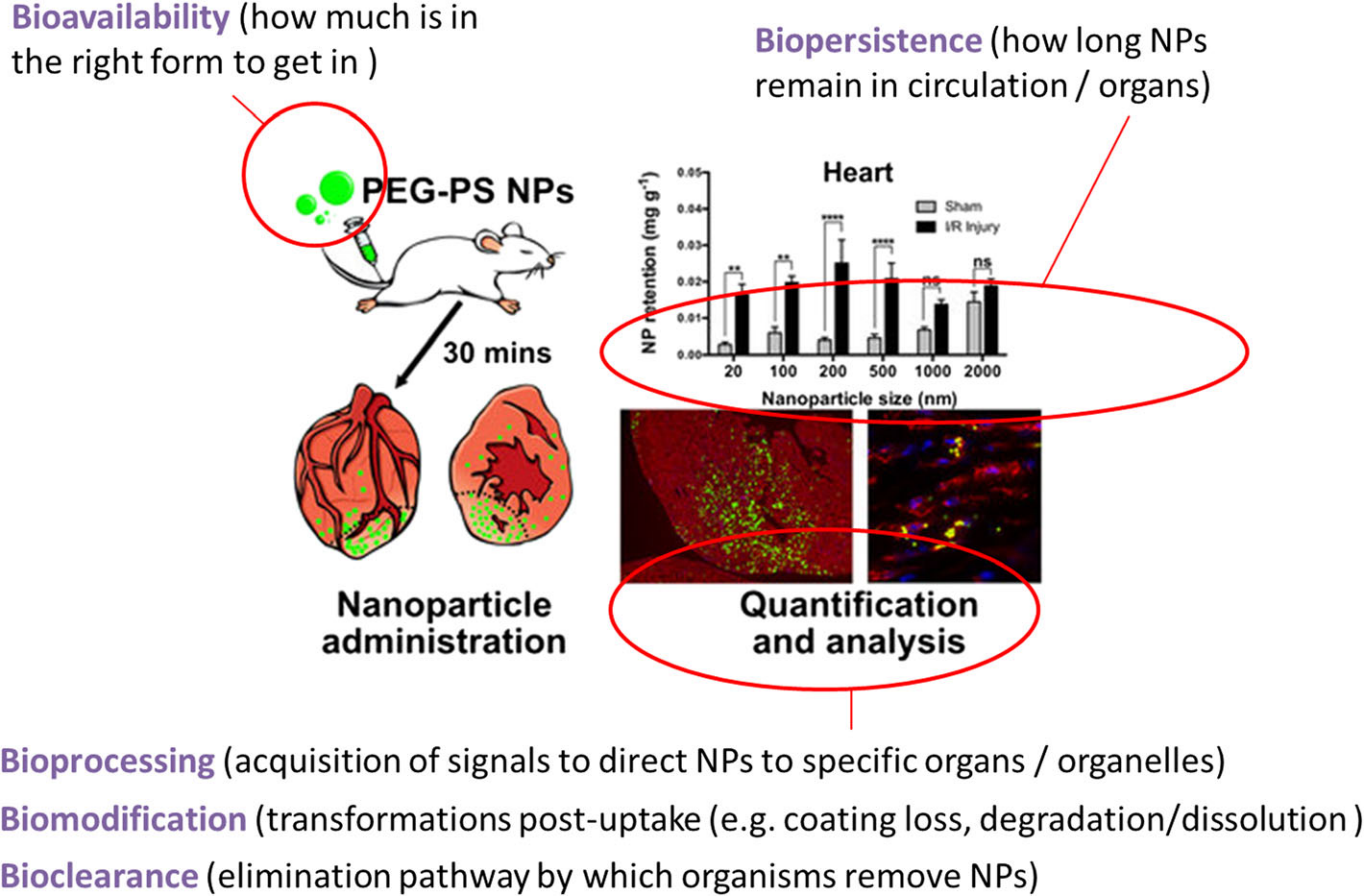
Conceptual understanding of the inter-relationships between the 5Bs and the working definitions of these terms as used in this section. Bioavailability indicates the amount of the applied dose that is in the right form to enter the organism, which for NPs depends on the dispersion conditions and the interplay between the medium components and the NP surface. Biopersistence provides an indication of how long the NPs remain in circulation and/or are retained by the organs to which they biodistribute (i.e. the retention half-life) as determined by their adsorbed biomolecule corona. Retention is affected by bioprocessing, which we define as the physicochemical transformation of the NPs by cells or organisms, which are often driven by the acquired biomolecules. Bioprocessing reflects the fact that NPs and their degradation products may impact on the biochemical functioning of the cell or organism, including assimilation into cellular reactions. Finally, bioclearance describes the elimination pathways by which organisms remove NPs, which are dependent upon the uptake route and the biodistribution pattern as different organs have different clearance mechanisms available, as well as the bioprocessing following localisation to the target organs.

It becomes clear from the snapshot of studies presented above that it is very difficult to untangle the 5Bs – they are interrelated and inter-dependent, but a clear understanding of each, and their combined impact, is vital for regulatory certainty. Fig. 6 provides an overview of our current conceptual understanding. There is a clear need for parallel *in vitro* and *in vivo* studies in the short term in order to untangle the pathways and mechanisms involved, with the *in vivo* studies in particular providing important insights into the final biomolecule coronas following *in vivo* biodistribution and/or during bioclearance [143]. While there is a strong drive to reduce reliance on animal testing, this can only be achieved once *in vitro* and *in silico* methods have proven to be predictive of *in vivo* health outcomes. Finally, it is clear that the adsorbed biomolecules play a central role in each of the processes underpinning NP bioavailability, biopersistence, bioprocessing including biodistribution, biomodification and bioclearance. Taken together, these studies suggest that there is still a major gap from fundamental science to regulatory relevance, but that progress is being made, and that the biomolecule corona may provide important insights and lead to the potential for predictive toxicology based on the bioprocessing signalling predicted from maps or fingerprints of NP-associated biomolecules. The formation of the biomolecule corona around NPs also raises the question of what is the relevant “form” to assess in regulation (e.g. the evolving NP-corona complex versus the pristine NP), especially as numerous studies show that bare particles are both more toxic, and rapidly acquire a biomolecule corona from their surroundings.

Future directions for research into the 5Bs include development of predictive models for NP corona formation and composition, and for corona influence on cellular attachment and uptake, biodistribution, bioprocessing and bioclearance, and as well as elucidation of key initiating events and the subsequent adverse outcome pathways related to biomodification resulting from exposure to NPs. From a biomedical perspective, significant research directions include understanding and controlling targeting of NPs to the desired location and the role of the biomolecule corona in driving this, as well as understanding and predicting the impact of the biomolecule corona on drug release rates and reducing off-target effects arising from sub-populations of the NPs being bioprocessed or biodistributed differently to the ideal. Significant effort is needed to elucidate the *in vitro – in vivo* correlations, especially given the very different biomolecule concentrations typical of each (10% versus >80% biomolecules, respectively), and the different biomolecule compositions in discrete tissue compartments *in vivo*, and the consequences of this for NP bioavailability, bioprocessing and bioclearance.

### Immunity and systemic responses

Nanosafety assessment needs simple and affordable, but at the same time robust and meaningful, assays. Readouts reflecting immune activation are in this context pursued by many groups [144]. This is reasonable, since the immune system has evolved to recognize non-self and to decide whether a defensive action is appropriate. In addition, immune cells are concentrated at the potential routes of entry for pathogens, which are the same routes by which NPs enter the body. This perspective will discuss what information can be gained when focusing on systemic rather than local immune responses.

Entry of a potentially dangerous non-self entity stimulates local reactions. If danger signals are sensed, a local inflammation ensues. The symptoms are familiar: Reddening and swelling (edema formation due to immigration of immune cells, stimulated by locally produced chemotactic factors), heat (from increased blood flow, to limit bacteria and viruses which often have a narrow temperature optimum), as well as pain and impaired function (limiting movement to avoid further injury and support wound healing). However, most contacts with non-self do not result in defence, due to the lack of danger signals. Still, the immune system will respond: Either with homeostatic fluctuations of no further consequence, or with the development of tolerance via several active mechanisms. The majority of immune responses indeed result in tolerance, which can be a source of misinterpretation. Nanosafety assessment requires distinguishing whether a response is merely adaptive or truly defensive, which can be addressed by the choice of endpoints. Most studies on immune effects of NPs have addressed the inflammatory (innate) immune response, with respect to local reactions [145–147].

Parameters that are characteristic for local responses are well detectable in many professional immune cells, but also in tissue cells which *in vivo* produce alarm signals to attract immune cells into a potentially threatened site. A good marker for local response is activation of the transcription factor NF-kB, which indicates cell stress that occurs during immune stimulation [45, 148]. The chemokine IL-8 is another example: It is produced very early and can be defined as a relatively unspecific alert signal, which indicates local inflammation that can be extreme enough to induce cytotoxicity [149–152]. These markers are readily measured and convenient for simple and affordable tests, but their use with not well characterized materials and cells requires care to confirm that an observed response is indeed defensive. In this respect, NPs present the challenges of heterogeneity and of batch-to-batch variation. Readouts like NF-kB and IL-8 are popular since they can be readily measured in cell lines and primary cells on the transcriptional and the protein secretion level, using methods like qRT-PCT, reporter genes and ELISA. However, , there is a risk of false positives when a normal homeostatic fluctuation in response to a stimulus is mistaken for an indication of danger to the body. Even worse, inflammation may be due to contamination, most commonly with the ubiquitous bacterial compound LPS [153] .

One way to deal with this problem is to look for parameters indicating systemic responses. For example, IL-1 is a major pro-inflammatory, fever inducing cytokine produced by several immune cell types, so detecting substantial IL-1 secretion would suggest a systemic inflammation which is certainly not indicating tolerance [154]. The related cytokine IL-18 shares many functions of IL-1 (but not fever induction) and offers the advantage that it is produced by numerous cell types [155]. Such readouts indicate a systemic inflammation that evolves from a local one: Think about flu that after a while affects the whole body, despite a very local occurrence of virus. The advantage of systemic inflammation markers is that they more clearly indicate a defensive reaction, thus immune mechanisms have assessed a stimulus as being dangerous.

Inflammation is often considered to be synonymous with innate immunity. This is an evolutionary ancient package of defensive mechanisms, which has the attractive feature that similar readouts can be made in invertebrates, offering opportunities to directly link human toxicology and ecotoxicology. In contrast, adaptive immunity has fully developed first in teleost fish and is thus limited to vertebrates. Adaptive reactions are always systemic, since they require the interaction of several cell types (especially antigen presenting cells, T-cells and B-cells) and involve secondary immune tissues, mainly lymph nodes and spleen. As in the case of innate immunity, a response is not the same as a defensive action. T-cells may be activated to proliferate and differentiate, but if they should be of the T_reg_ type (regulatory T-cells), they will produce immunosuppressive cytokines and thus promote tolerance.

Adaptive immune responses can develop in three directions, of which the “default” is tolerance. Type 1 and type 2 responses are defensive, the first being directed mainly against bacteria and viruses, the second mainly against macroparasites. Both are distinguished by their cytokine pattern, especially for the cytokines produced by T-cells. IFN-α and IL-4 are among the tell-tale markers for type 1 and type 2 responses, respectively. A functional definition can be made via the isotypes of antibodies that are produced by B cells. IgG_1_ is the most prominent antibody type in blood and it increases substantially during type 1 responses. Since adaptive reactions develop more slowly than innate ones (days vs. hours), it makes sense that type 1 effectors like IgG_1_ interact productively with innate immune mechanisms. Assessing type 1 responses is more challenging than testing for inflammation and cell stress, since a full response can be mimicked only in co-culture systems rather than in single cells. Some NPs have been shown to influence the development of antibodies, even though they are themselves only rarely recognized by antibodies [156].

Type 2 responses are associated with parasites, but are for many people more familiar as a pathophysiological response in allergic diseases. IgE antibodies mediate these responses. They have very low levels in serum, but are bound to high-affinity receptors on the surface of effector cells (basophils, mast cells, eosinophils). So far, no case has been described where NPs act as allergens [157], but it has been shown that binding of allergens to NPs can enhance their allergenicity [158].

So far, most studies have investigated immune effects of NPs as single agents. In the future, we can expect an increasing number of studies that treat NPs as one component in a complex exposure situation, which corresponds more closely to real life. In addition, NPs can have effects on the development of a systemic antibody-mediated response, but it is not well known under which circumstances and to which extent this takes place in the body. Nanomedicine can address these issues more easily, since it deals with high concentrations and controlled exposure. Developing methods that allow considering adaptive immune reactions for risk assessment is at present a challenge. The effects of co-exposure to NPs and to stimuli that induce either systemic type 1 or type 2 responses will increasingly become a subject for nanoimmunotoxicology research aiming at risk assessment.

### Non-Specific Effects of Particles

As non-specific effects of particles, mixed dust pneumoconiosis and response of autonomic nervous system to exposure to particles are discussed.

#### Mixed dust pneumoconiosis

Specific effects of particles can be typically observed in silicosis and asbestosis. Silicosis and asbestosis show the very special features of pneumoconiosis. The silicotic nodule is one of the characteristic histopathological changes observed in the lung during silicosis [159], with large areas of opacity being one of the typical radiographic patterns observed in silicosis. In silicosis, small opacities are dominantly distributed to the upper lung field, and in asbestosis, small opacities are mainly distributed to the lower lung field. Pleural changes can be specific to asbestosis, whose histopathology shows interstitial fibrosis and formation of asbestos bodies.

On the other hand, the mixed dust pneumoconiosis might be one of the non-specific types of pneumoconiosis [160]. It is important to note that there is no full consensus on a definition for mixed dust pneumoconiosis. Nevertheless, several reports showed that similar histopathological features, which are different from silicosis or asbestosis, are associated with exposure to different kinds of dust. These suggest the existence of non-specific effects from different kinds of particles in humans. Small rounded opacities are specific to silicosis, while small irregular opacities are observed in asbestosis or mixed-dust pneumoconiosis. Mixed dust pneumoconiosis is found in foundry and welding workers [160] or coal miners, who are exposed to dust containing less crystalline silica. Contrary to silicosis, mixed dust pneumoconiosis produces mixed dust fibrous nodules characterized by a stellate shape of the nodule. According to the Honma in Japan, the causative agent for mixed dust pneumoconiosis is non-fibrous silicate plus a low content of free (crystalline) silica. The characteristics of X-ray findings of this pneumoconiosis are irregular opacities and ill-defined small rounded opacities. Histopathologically interstitial fibrosis and fibrotic stellate nodules are observed in mixed dust pneumoconiosis. Lastly the prevalence of this disease is 34% of the entire pneumoconiosis [161]. There are case reports suggesting that a variety of particles may induce mixed dust pneumoconiosis [161]. Pathology shows mixed dust nodules outnumbering the silicotic nodules consistent with mixed dust pneumoconiosis. Graphite was detected in biopsy samples using laser microprobe mass analysis [162]. The sample was obtained from a foundry worker with mixed dust pneumoconiosis. While the prevalence or incidence of silicosis has been decreasing, there are concerns with pneumoconiosis which may be induced by different kinds of particles other than silica. Mixed dust pneumoconiosis may be one such pneumoconiosises. Although an epidemiological study showed less excess risk of cancers in mixed dust pneumoconiosis than in silicosis, there are still concerns with possible cancers or autoimmune disease resulting from the mixed dust pneumoconiosis.

#### Effects of particles on cardiovascular or autonomic nervous system

A recent study on effects of exposure to titanium dioxide particles suggested the cardiovascular or autonomic nervous system as being among the possible non-specific targets of particles [163]. Preliminary investigations in China suggested high exposure of TiO_2_ to the workers in this specific factory. The study investigated the respiratory and cardiovascular status of the workers who were expected to be exposed heavily to TiO_2_ particles to find any possible adverse effects from TiO_2_ particles. The number of particles with diameter less than 300 nm was estimated using two different mobile instruments – condensation and optical particle counters (CPC and OPC, respectively). Although there may be problems theoretically to use the difference in the number between CPC and OPC as shown, it is believed that this can be used as an indicator reflecting the number of small particles. The primary particles were traced to measure their diameter. The diameter of the majority of primary particles was more than 100 nm, but certainly part of the primary particles are less than 100 nm in diameter. The analytical SEM analysis showed that the particles were titanium dioxide. Distributions of titania and oxygen completely merged on the image of particles. Mass based concentrations ranged from 18.6 to 30.8 mg/m^3^. Compared to the first investigation, which showed the concentration of titanium dioxide to be more than 100 mg/m^3^, the concentration of titanium dioxide in the last investigation was reduced, by improvement of the facilities in the factory. Multiple regression analysis showed that heart rate was positively associated with the number of small sized particles with diameter less than 300 nm. Inversely, RR50, the frequency of serial heart beats fluctuating more than 50 msec, was negatively associated with the number of particles less than 300 nm. As the RR50 is a parameter of parasympathetic function, it is considered that exposure to small particles of titanium dioxide suppresses parasympathetic function. The association between the parameters of heart rate variability and the number of small particles of titanium dioxide was also analysed at different delay times. The number of nano-scaled particles showed significant positive association with the total number of normal to normal heart beat rate (N-N) and negative association with the mean N-N interval, RR50+, and percentage of RR50 +/- 1 min delay. Multiple regression analysis on the pooled data of the four workers shows that particle number less than 300 nm in diameter were associated positively with the heart rate and negatively with the percentage of RR50, which is a parameter of parasympathetic function. The present study generated the hypothesis that exposure to particles affects autonomic function in workers handling TiO_2_ particles. Other studies on heart rate variability show similar results in terms of suppression of parasympathetic functions with different kinds of particles, suggesting non-specific effects of particles.

The overall conclusion is that the idea of mixed dust pneumoconiosis suggests the existence of non-specific effects of particles, which might be involved with pneumoconiosis. Possible adverse consequences, including cancer, autoimmune disease or cardiovascular disease, from non-specific effects of particles should be further investigated. As one of non-specific effects of particles, acute effects on the autonomic nervous system should be further investigated, through epidemiological or experimental studies.

### Particles and the Developing Body

Understanding the developmental toxicity of NPs is vital because exposure to fine particulate matter during gestation increases the risk of low birth weight in the child [164], which is associated with health and disease later in life [165, 166]. Recent clinical cohort studies suggest that prenatal and neonatal exposure to fine and ultrafine particulate air pollution is associated with an increased risk of developmental brain disorders, such as autism spectrum disorder and schizophrenia, in offspring [167–169]. It is important to understand the mechanism of action underlying the effect of NPs on the developing body in order to reduce the toxic risk of these atmospheric ultrafine particles and to promote the safer use of engineered NPs for future generations.

We start to have some understanding of the mechanisms underlying the potential hazards of fine and ultrafine particles to brain and behaviour. The effects of particle exposure can be studied using brain perivascular histopathology [170]. In particular, the expression levels of proteins associated with astrogliosis, e.g. glial fibrillary acidic protein, may be useful as a sensitive and quantitative marker of maternal exposure to low doses of NP for prediction of their developmental toxicity [171]. There seems to be potential protective effects of antioxidants on the brain perivascular abnormality (astrogliosis) of offspring mice whose mothers were exposed to carbon black NPs. One of the anti-oxidants, N-acetyl cysteine, partially suppressed the astrogliosis in the brain of offspring induced by maternal exposure to carbon black [172]. There are also developmental effects of perinatal exposure to experimental secondary organic particles as prepared by oxidation of diesel exhaust particles with ozone. Neonatal mice exposed to secondary organic particles demonstrated a decrease in social behaviour with down-regulation of estrogen receptor-β and oxytocin receptor in the hypothalamus [173]. Ming-Wei Chao et al. reported an increase in reactive oxygen species and several cytokines in the amniotic fluid, and changes in microRNA expression profile in the cerebral cortex and hippocampus of foetal brains in rats following exposure to PM_2.5_ [174].

Recently, the potential use of zebrafish (*Danio rerio*), Drosophila, and chicken embryos has been investigated as alternative methods for high-throughput screening of the developmental toxicity of NPs [175]. The zebrafish model, in which hatching rate, developmental malformation of organs, genotoxicity, immunotoxicity, abnormal behaviour, and neurotoxicity can be measured, is useful in the field of toxicology and biomedical research to evaluate the reproductive and developmental toxicity of NPs [176]. The advantages of the zebrafish model are their small size, high reproducibility, quick development, and transparency of the embryo [177]. The transparency of the embryo enables observation of all the cells from early larval stages and facilitates real-time imaging of NP distribution *in vivo* during the embryonic development [178]. In addition, their genetic information is accumulating rapidly by genome sequencing [179]. This model has also allowed comparative analysis of the developmental toxicity of NPs with different properties, such as size [180, 181], chemical composition [182, 183], and surface modification [184]. On the other hand, the chicken embryo model has an advantage that the direct effect of NPs on the embryonic development can be evaluated by removing the indirect effect mediated by maternal factors easily, as reported for the results of developmental toxicity of TiO_2_-NP [185] and carbon NPs [186–188]. In addition, the use of Drosophila, which has a short life span and 77% of the human disease genes [189], has just started in terms of developing cost-effective high-throughput screening methods for assessment of the developmental toxicity of NPs [190]. These animal models may provide rapid hazard assessment techniques to facilitate regulation and ensure safer NPs reach the market thereby protection future generations.

Previously, the translocation of NPs from maternal to foetal circulation, with ultimate deposition in the offspring’s body, was first reported in an experimental model where TiO_2_ NPs were subcutaneously injected into pregnant mice [191]. Experiments with an *ex vivo* placental perfusion model showed an inverse correlation between the translocation of nano- and submicro-particles (in this case, polystyrene beads) and particle size [192]. Recently, the dose- [193] and size-dependency [194] of NP translocation from the mother to foetus was also reported in *in vivo* studies. Overall, NP translocation through the placenta is likely dependent on particle surface modification [195, 196], chemical composition [197], and the timing of the exposure during pregnancy [196]. Because the mass-based translocation ratio is not high, the detection of these particles is dependent on the analytical techniques used. It is important to understand not only the direct effect of NPs on the foetus, but also the indirect effects mediated by circulating cytokines or other secondary messengers generated from oxidative stress and pulmonary inflammation in the maternal lungs [198–202] [174], placental dysfunction [199], or genotoxicity [203, 204].

The reproductive [191, 205–210] [211], immune [200, 212, 213], and central nervous systems have been investigated as potential targets of maternal NP exposure. In particular, the effect of this exposure on the offspring brain is getting better understood. While evaluation of developmental neurotoxicity via histopathology [170, 214, 215], and determination of DNA methylation level [216], monoamine level [217–220], and expression profile of mRNA [221, 222], X-chromosome inactivation factor [223] and microRNA [174] is valuable, evaluating the neurobehavioral changes in children after prenatal NP exposure is also important in understanding the developmental neurotoxicity of NPs. Previous studies have shown that exposure to NP-rich diesel exhaust or concentrated ambient particles (CAPs) during prenatal and/or neonatal period altered spontaneous locomotor activity level [217, 224, 225], decreased novel object recognition [226] and motor coordination [218], and increased autism-like repetitive and impulsive behaviours [167, 224]. Moreover, maternal inhalation studies showed that exposure to nanoparticulate TiO_2_ during the foetal period decreased visits to, and duration of, stay in the central zone during evaluation with the open field test in mice [198] and impairments of working or short-term memory and initial motivation in rats [227]. A decrease in spontaneous locomotor activity in a novel environment was also reported in mice whose mothers were exposed to carbon black NPs [201]. The sex difference in response to nanoparticle exposure during the fetal/perinatal period is likely important [167, 198, 201, 225, 228]; however, further investigation is needed to clarify the mechanism of the difference.

Histopathology suggests that brain perivascular cells, including perivascular macrophages and their surrounding astrocytes, have an important role in clearing waste from the brain parenchyma [229, 230]. They can serve as an extremely sensitive marker of maternal exposure to low doses of NPs for prediction of their developmental toxicity [170]. The histopathological changes were confirmed with decreased number of parvalbumin-positive interneurons following a maternal inhalation study [231]. Sensitive and quantitative endpoints evaluating the developmental impacts of maternal NP exposure are necessary to facilitate the risk assessment and hazard identification of NPs [232]. Future work will hopefully clarify the mechanism of developmental toxicity of NPs, and aid in the development of preventive strategies against intended and unintended NP exposure.

### Particles in the natural environment and links to human health

#### Hazards from Natural particulates and the evolution of the biosphere

The founding assumption from the perspective of respiratory toxicology is that airborne particulates are deleterious to human health. This notion is supported by decades of scientific research (see above). However, this does not mean that all particulates are, by default, toxic to humans or any other organism. The geochemical cycles on our planet provide many different natural sources of particulate materials, a few of which are hazardous to health. For example, acute airborne exposure to particulates from volcanic activity [233], the soot from natural forest fires [234], or sand from Sahara dust events [235] can cause respiratory distress and subsequent adverse effects on the cardiovascular system.

These ultrafine particulates from natural sources were directly effecting human health over evolutionary time periods. It is also possible that geological events and/or extreme weather events had an indirect long-term effect on human health via an impact on ecosystem services (see below). These natural particulates are produced in billions of tons annually, with particles from deserts alone estimated as upwards of 500 million tons per year to several billion [236], and from the view point of risk, the number of deaths attributed to respiratory exposure to these natural particulates is hard to estimate given that pollution is a mixture of substances and with many confounding factors in the health indices used [237]; but might be of the order of ten extra individuals/1000 deaths in the local population. In comparison, today natural disasters account for some 375 million deaths per year [238]. From an evolutionary view point, both examples are a small fraction of the 7.5 billion people on the planet.

Nonetheless, climate change has raised concerns about the increased frequency of adverse weather events, including air pollution. The immediate consequences may be an increased death rate or other adverse health outcomes on the exposed population. For example, the annual mortality attributed to PM2.5 is around 2.1 million deaths, but past climate change may account for approximately 2,200 annual deaths from PM2.5 [239]. The contribution of climate change to air quality deaths is a few percent of the total. One might argue that natural exposure to airborne particulates, climate change, etc., are simply ongoing selection pressures on human evolution.

Environmental change may simply select genotypes from the human population that are more resilient. The notion that all life on Earth has evolved with the geosphere is well established [240], with key geological events influencing both the biodiversity of the planet and also new biochemical adaptations that enable the organism to survive. Even relatively small events in the context of geological time have altered the rates of divergence in the human gene pool (e.g., the last ice ages [241]). There is evidence that exposure to naturally occurring particles can infer resistance in future generations. In the laboratory, this phenomenon is demonstrated with organisms with short generation times. For example, exposure of multiple generations of the microbe *Pseudomonas aeruginosa* to natural minerals of silica, anatase TiO_2_ or alumina informs on a resilience to the minerals arising from adaptation of the genes controlling the extracellular polymeric substances that are secreted as a protective barrier by the organism [242]. The evolution of resistance to engineered NPs has also been recently demonstrated in the microbe, *E. coli* exposed to silver NPs [243]. For microbes in the laboratory with a generation time of ~ 30 minutes, resistance may become apparent within one day, or some 40+ generations. The human genome has also shown resilience to ‘new’ forms of organic particulates. Viral-like particles are estimated to have been introduced into the primate genome some 20 million years ago, and humans have evolved defences against such viral-like particles or retrotransposons, which are now regarded as a sub-type of the endogenous retrovirus [244], that comprise an estimated 3-5 % of the human genome. Of course, with an average life-span of some 77 years, any apparent resistance to new particulate exposures now would take the next 3000 years to manifest as a genetically resistant strain of humans. The climate record has changed significantly since the industrial revolution, and predictions suggest that warmer conditions in the next fifty years may increase wind-driven erosion of soils (e.g. [245]), speeding up the geological process of generating PM10 (PM smaller than 10 μm) and other dust. The dilemma is that for the first time in human evolution, the rate of change in particle release from the geosphere may outpace our genetic ability to adapt.

#### Environmental exposure to particulates and human health effects

The relationship between exposure and effect is relatively well-known for airborne particles. The lung is a critical target organ with the penetration of the material into the airway being dependent on particle size. The greatest concern is with regard to ultrafine particles that may penetrate into the alveolar space, and epidemiology studies have demonstrated clear respiratory health effects with PM10 and PM2.5 particles (PM smaller 2.5 μm). The resulting lung inflammation and respiratory distress will also alter cardiovascular function as the body attempts to maintain constant ventilation-perfusion ratios. Consequently, elevated blood pressure and increased hospital admissions from heart disorders is a common feature of air pollution events. There may also be direct cardiotoxicity from gases in the polluted air such as carbon monoxide.

In stark contrast, understanding of how incidental dermal or oral exposure to particulates in the environment affects human health is more difficult to establish. The keratinised nature of human skin with the triple layer of epidermis, dermis and hypodermis is regarded as an effective barrier to substances in the environment, provided that the skin remains intact. Exposure to airborne soot particles, or particulates from traffic pollution, are partly associated with ageing of the skin and altered pigmentation [246]. Air pollution has also been implicated as a cause of skin cancer, where the particulates may act as a delivery vehicle for carcinogens such as benzo-a-pyrene (BaP) [247]. The subsequent oxidative stress from BaP is proposed to initiate inflammation of the skin; eventually leading to DNA damage.

The gut is a rather different mucous barrier, consisting of mucous epithelium, sub-mucosa, the underlying muscularis (longitudinal and circular muscle) and the serosa. Oral exposure of the general public to substances via the food is largely managed via guidelines for soil quality, as well as limits for the allowable contaminant residues in crops, livestock, fish and shellfish. There are of course, quality standards for food products arriving on our supermarket shelves. There are some international agreements on food safety standards, listed in the CODEX *Alimentarius* ([248]. These regulations are aimed at specific types of food products. For example, to regulate the allowable *Salmonella* concentration in raw meat, mycotoxins in cereals, or the amounts of individual chemicals that may be used as a food additive. There is also national level legislation, and for example in the U.K., the Foods Standards Agency has responsibility for food safety. The legislation on food safety is often intended to relate to a process or a health outcome. In the Food Safety Act (1990) in the U.K. [249] the offences are with regard to injury to health, introducing products into the supply chain that are not fit for consumption, or misrepresentation of the food product. Thus there are no specific provisions for individual chemicals or particulates *per se*; but an adverse health effect caused by a particulate in food (or any other material) would be covered by the legislation. NPs and micron-scale particulates are not listed in standards relating to food contaminants or food additives at this time. In essence, none of the current food regulations consider particulates. Nonetheless, there are limits for toxic substances such as mercury in fish and shellfish. For metals at least, one concern is whether or not the existing standards might also protect the public from the nano form. This is problematic for several reasons. Firstly, the gut is able to differentiate crystal structures of the same chemical substance (e.g., TiO_2_ materials [250]). Thus any standard would need to consider shape and size. Secondly, the regulations and standards are intended to give a margin of safety below the no effect concentration on human health. The gastrointestinal tract is the route of exposure, and yet, even for this body system the effects of NPs on gut functions (motility, secretion, absorption and digestion) are largely unknown.

One possible exception with respect to protection from oral exposure to incidental particles is within the standards for drinking water. For example, in the U.K. the Drinking Water Inspectorate enforces water quality at the tap for turbidity (< 4 NTUs) and colour (limit of 200 mg/L Pt/Co). It is conceivable that a particulate material in the water may be limited by these measurements of water quality. However, the turbidity and colour is determined mainly with respect to clarity and appearance of the water, rather than any chemical hazard to the consumer. Of course, some substances in drinking water are likely to be colloidal such as iron derived from the piped supply or the natural organic matter in the water, but there is very little information on particulate hazards to human health through drinking water supply. Legacy contaminants such as asbestos fibres have been found in drinking water supplies, with some 40 cities have concentrations exceeding 1x10^7^ fibres/litre ([251]. However, revealing cause and effect is challenging. Gastrointestinal tract cancer rates maybe higher in cities that also have elevated asbestos in the supply, but how much of this can be attributed to asbestos compared to other risk factors such as occupation, diet, smoking and alcohol consumption etc., is unclear [252].

Engineered NPs have become a specific concern with respect to the human food chain [253, 254], and also drinking water supply with the potential applications of nanotechnology in disinfectants and filtration systems [255]. One of the technical challenges ahead is the development of routine detection methods for NPs in environmental samples [256], and although some methods have been recently developed such as single particle induction couple mass spectrometry sp-ICP-MS, there remains reliance on computational models to predict environmental concentrations of NPs, e.g., [257]. The concentrations of NPs predicted in surface waters in the EU are typically at μg/L levels [258], and the main food chain risks appear to be via the application of NP-contaminated sewage sludge to agricultural soils. However, in comparison to substances like mercury, the technical knowledge on the fate and behaviour of NPs through aquatic or terrestrial food chain to humans is limited. The many knowledge gaps on environmental fate contribute to uncertainty such that a reliable human health risk assessment with respect to incidental exposure via the environment is not possible for most NPs. Notably, agreement has yet to be reached on bioaccumulation tests for NPs [259]. Bioconcentration- and biomagnification-like factors for NPs are currently lacking with respect to the transfer through trophic levels to humans.

#### Particulates, ecosystem services and indirect effects on health

Human health is also affected by the quality of our ecosystems and the biodiversity therein. Food supply, water security and habitable living space are significant pressures that may ultimately limit the human population. The notion of protecting ecosystems so that they provide essential services; such as the ability to grow food, clean groundwater and recreational amenity is now well-established [260, 261]. The health benefits of growing crops on uncontaminated soil, or abstracting drinking water from a pristine lake are apparent. However, the amenity value of green spaces and/or the coastal zone to human health are also important in terms of exercise/cardiovascular health, mental health and general wellbeing [262]. From the view point of particle exposures, the short-term outcome of air pollution may be the temporary loss of these outdoor amenities, as well as some limited contamination of the soil, crops and surface waters. The chronic impact of particulate exposures to ecosystems and the subsequent indirect human health impact as a result of adverse effects on ecosystem services is less clear. Diffuse atmospheric inputs of dust from soil erosion might be argued to adversely affect crop production and food supply, but the deposition of dust is also part of the ongoing geological process of making new soil. The issue is whether a spatial or temporal change in this cycle, or a change in the turnover of geological processes, impacts on human health via the ecosystems involved. There is some evidence that air pollution impacts the ecosystem services provided by forests [263]. However, contribution of the particulate component in the air pollution to such ecological impacts, and subsequent quantifiable changes in human health are not yet determined.

### Lessons from the past for future toxicological studies on big, small and variously shaped particles

#### Mining and Asbestos: the first two challenges in particle toxicology

Mining has been not only the oldest but also the largest single industry where most dust-related occupational diseases were described. Human populations in these dusty environments have therefore provided the first line of evidence on the existence of a correlation between exposure to dust and the diseases observed. Countries where such mining activities have existed were the first to report on these diseases by centres dedicated to conduct such research. For example, in South Africa, mining for a number of commodities including gold, asbestos and coal have existed since 1886 when gold was first discovered and was fully commercialized in 1911. This has necessitated instituting the first Miners Phthisis Act for pre-employment and health examinations and opening of the Miners’ Phthisis Bureau in 1916 for pre-employment, periodical and compensation examinations of miners for pneumoconiosis [264]. The existence of considerable deposits of crocidolite, amosite and chrysotile asbestos in South Africa and their commercialization has prompted the study of asbestosis as early as 1926 where its pathology was studied [265, 266]. Coal deposits of anthracite and bituminous type in the country also made it possible to report on pneumoconiosis in this mining industry [267]. In response to the necessity to conduct research in dust-induced occupational lung diseases in these different mining industries, the Pneumoconiosis Research Unit was established, presently known as the National Institute for Occupational Health (NIOH), by the mining industry and by the Department of Mines in 1955. Coal mining was also an important industry in UK and the USA and hence similar activities were taking place in Cardiff, United Kingdom (UK) and in West Virginia, USA where pneumoconiosis was an issue in this industry [268, 269]. In the UK, once again, out of necessity for further research, the Edinburgh Institute of Occupational Medicine (IOM) in UK was founded in 1969 as a charitable research institute by the National Coal Board to research mining diseases [270] and in the USA, the National Institute for Occupational Safety and Health (NIOSH) by the USA congress by passing the Occupational Safety and Health Act in 1970.

It is of importance to document that these and other institutes were instrumental in producing sentinel publications and also hold a number of conferences on particle-induced diseases. For example, the South African Institute hosted the first pneumoconiosis conference in 1930 where asbestosis was acknowledged as a new occupational disease [271]. The two next conferences on pneumoconiosis were again hosted by the South African Institute in 1959 and also in 1969 where important issues were discussed ranging from dust measurements, dust composition to the pathology of asbestosis, silicosis and cancer in humans and in animal experimentations. It was at the conference in 1959 where Wagner provided definitive evidence of the etiological association between asbestos exposure and mesothelioma cases in the mining industry in South Africa [272, 273]. The production of mesotheliomas in animals (Wistar rats, mice, and guinea pigs), was also confirmed with experimentation by injecting various forms of asbestos (crocidolite , amosite, and chrysotile, and carbon black) into the pleural cavities where mesotheliomas could be produced mainly from crocidolite asbestos [274, 275]. In the UK, the association between coal dust exposure and risk of pneumoconiosis was confirmed by scientists at IOM in the UK with a landmark paper published in 1970 [276].

Although during this early period the investigations were more centred on the pathology of pneumoconiosis, some attempts were also made to study the parameters involved in the toxicity and pathogenicity of mine dust. As early as in 1913, it was shown that the size of the particles that could have access to the lung proper had a maximum diameter of 10 μm [277]. Questions were then posed as to which size of mine dust needed to be measured as it was realized that dust hazard in the mining industry was not the average dustiness of the whole mine but what kind of dust and what size of dust have caused pneumoconiosis [278]. With animal experiments, it was also shown that smaller particles were the most dangerous in the production of silicosis [279, 280]. The effect of the crystalline nature of silica particles was also tested in relation to the severity of pathogenic reactions and it was found that tridymite was most pathogenic followed by cristobalite, quartz, and fused silica [281]. At the second International meeting on pneumoconiosis held in 1969, special attention was paid, in addition to size, to composition and shape of asbestos fibres [282]. Subsequently, the importance of the three to one aspect ratio in the pathogenesis of inorganic fibres became known as the Stanton hypothesis [283, 284].

With the realization that the physicochemical properties of asbestos fibres were of importance in producing their pathogenic effects, recommendations were made by the Working Group on Asbestos and Cancer at a special meeting in New York in 1964 under the auspices of the Geographical Pathology Section of the Union for International Cancer Control (UICC), to prepare standard samples for different commercial asbestos types for international inter-comparative research experimentations [285, 286]. On this recommendation, half a ton of the five main asbestos: Rhodesian chrysotile (Chrysotile A), Canadian chrysotile (Chrysotile B), South African amosite, Finnish anthophyllite, and South African crocidolite, were prepared in South Africa. These samples became the well-known UICC Standard Reference samples for animal experiments [287–289].

It was also demonstrated, as early as in 1960 that the surfaces of silica have peroxidative activity where this activity was shown to be increased with the presence of trace amounts of iron and further increased with the addition of hydrogen peroxide. It was then speculated that a substance is released with high peroxidative properties and that there appeared to exist a correlation between the oxidative activity of the dusts and the known fibrogenic potential of the dust materials examined [290, 291]. These authors suggested that the reactions induced by quartz are catalytic ones with reactions involving free radicals similar to those suggested previously by Johnson et al [292]. The observations that quartz powder possesses oxidative and hydroxylative properties suggested that reduced glutathione might also be affected by quartz [293]. Furthermore, the protection afforded by reduced glutathione to macrophages incubated *in vitro* with tridymite and etched quartz has suggested that the toxic actions of these dusts might be exerted among other things, through changes in the sulfhydryl-disulfide form [294]. Surfaces of silica were also shown to have adsorptive properties with the ability to adsorb dyes, amino acids, proteins, and metal hydroxides [295]. Such adsorptive properties were also shown for the surfaces of asbestos fibres to adsorb carcinogens such as 3,4-benzpyrene [296]. It was later proposed that this carcinogen from cigarette smoke adsorbed onto the surfaces of asbestos fibres may enhance the presentation of this carcinogenic compound to cellular constituents and thus play some part in the overall biological activity of the inhaled fibres [297]. On the other hand, the possibility of the release of components from some mineral fibres could also be shown in relation to their toxicity [298].

It was subsequently hypothesized that if the surface layer is in fact an important parameter for silica, its inactivation may alter particle fibrogenicity. Surface pacifying agents have included metals and organic polymers. For example, aluminium and aluminium oxides were already extensively used as therapeutic agents for silicosis in North America and elsewhere [299] with no unanimity of opinion about the effectiveness of this treatment [300]. It was also noted that the toxicity of silica particles towards phagocytes in tissue culture can be prevented or reduced by the addition of small amounts of nitrogenous bases called as “compound 48/80” [301]; when tested *in vitro* [302] or injected intravenously this compound gave some measure of protection against intravenously injected silica in mice [303]. It was postulated that both “compound 48/80” and aluminium may have acted by changing the surface properties of silica rendering it less toxic [295]. However, its toxicity to humans [304] prompted the investigation of other similar polymers including compound 46-107 [302] and poly (2-vinyl-pyridine) or poly (4-vinylpyridine) [305]. Later, the latter authors oxidized these polymers with hydrogen peroxide in acetic acid to produce the soluble form poly (2-vinyl-pyridine­N­oxide) [306], which was proposed to act via its ability to coat the surface of silica dust [306]. This was later hailed as one of the most promising advances in the field of pneumoconiosis [307]. Other chemicals tested have also included dimethyl dichlorosilane which was thought to combine with the surface OH-groups. Histological examination of the lesions formed with animal experiments did not however show any significant difference when compared with a control series in which uncoated quartz was used [308]. It was agreed that this may have been due to the hydrolysis of the compound from the surfaces of the silica particles [309].

These early investigations laid foundations for particle toxicology with further determination to find answers to the central question: what made a particle or a fibre toxic and pathogenic? Significant activity in this field has produced impressive results which were presented and discussed at a series of particle toxicology meetings. Presentations made at these meetings were true reflections of the type of particles investigated and the important physicochemical properties of particles and fibres which determined their toxicity and pathogenicity. Most importantly, progress was made over the years in elucidating the mechanisms involved in this toxicity and pathogenicity. At the 7^th^ particle toxicology meeting in 1999, the ambient particulate matter (PM_10/2.5_) was first discussed, as at the previous meetings up to 1996, the particles discussed were exclusively silica and asbestos [310]. True to the predictions by these authors that PM issues would follow the same route as asbestos in a decade or so as other issues will come up, and with the publication of articles recognizing the new discipline of Nanotoxicology [77, 311], NPs became the subject of discussion and pointed to the importance of applicability of the earlier elucidated mechanisms and the established physicochemical properties from other particles to NPs. Describing the progress made over the years in particle toxicology will only serve in preventing to waste valuable resources by repeating what has already been achieved and will generate new knowledge based on what has already been established during the last four decades of particle toxicology.

#### Lessons learned from particle toxicology for future Nanotoxicology

The early observations in the mining industry in early 1900s up to late 1970s between exposure to particles and fibres and the diseases they have produced have prompted investigators to conduct *in vivo* animal and *in vitro* cell culture studies [312–314]. The next three decades work continued in earnest for the identification of the particle and fibre properties as well as in the elucidation of mechanisms involved in their toxicity and pathogenicity.

The decade between 1980 and 1990 produced an impressive number of investigations on the importance of the physicochemical properties of particles and fibres in producing toxicity and pathogenicity. These have included once again size [315, 316] and also crystalline nature [317], adsorptive ability of alveolar and serum components as well benzo(a) pyrene [318–321], and dissolution and biodegradation of mineral fibres [322, 323]. Moreover, surface reactivity with the generation of free radicals [324–328], and their ability to induce the peroxidation of cellular lipids were also studied [329–334]. But most importantly, these investigations have included the elucidation of the role of iron in these processes [335, 336] and also the importance of surface properties in the toxicity of particles and fibres with subsequent reduction of this toxicity through surface modification [321, 337]. The involvement of inflammation and inflammatory cells with subsequent production of cell-mediated reactive oxygen species and inflammatory markers in the toxicity of particles and fibres was also investigated [338–345]. In addition, the involvement of active oxygen species as secondary messengers for toxicity was investigated [346] and the prevention of such toxicity by scavengers of active oxygen species was also presented [347]. Finally, oxygen free radicals and oxidative stress and other mechanisms involved in the carcinogenicity of particles and fibres were systematically pursued [348–351] and assessment of the levels of antioxidant parameters were proposed as biomarkers of particle-induced diseases [352].

The decade between 1991 – 2000 continued to emphasize the importance of physicochemical properties of particles and fibres where the importance of surface area [353] and dissolution [354] in their toxicity were emphasized. Moreover, based on the surface adsorptive property of particles and fibres, a reduction of toxicity could be achieved by coating them with large molecular weight organic materials [355] and that differences in this adsorptive property correlated with differences in their toxicity [356]. The generation of free radicals and inflammatory cytokines by phagocytic and other cell types was continued to be investigated [357–360] and the relationship between this property and their toxicity and pathogenicity was established [361–364] where once again the reduction in toxicity with the administration of antioxidants was demonstrated [365]. Particle and fibre-induced free radical production of peroxidation of lipids and damage to DNA was also shown [366–369] where the role of iron, once again, in this damage could be demonstrated [370–377]. In addition, the importance of not only total iron present but also the type and oxidation state could be shown [374, 378–380]. Signalling pathways, gene expression, and cytokine production have also featured prominently in the effort to elucidate particle and fibre induced mechanisms of disease including fibrosis and cancer [381–386] and subsequently the expression of these parameters was considered to be an appropriate biomarker for measurement following exposure to particles and fibres [387–389]. Finally, the importance of biopersistence of mineral fibres in their pathogenicity and carcinogenicity was emphasised [390].

The decade between 2001 and 2010 was also very productive especially in elucidating the importance of surface properties of particles and fibres and their variability in relation to their biological effects [391–404]. Subsequently, efforts were presented whereby changes to these surface properties were introduced to reduce their toxicity and pathogenicity [405, 406]. Elucidating the mechanisms of disease through signalling pathways and gene induction have also produced landmark publications [407–413]. Finally, additional properties for ultrafine particles were described including their ability to translocate to other organs from their original route of exposure [414, 415].

Over the last three decades, particle toxicology could therefore successfully contribute to the identification of physicochemical properties of particles and fibres that may determine their toxicity and pathogenicity. These have included size with ability to translocate, crystalline nature, and surface properties including surface area, ability to adsorb macromolecules and ability to release ions through dissolution, surface activity through their ability to generate acellular as well as cell-mediated free radicals to produce the peroxidation of lipids and induce cellular oxidative stress. Suggestions were therefore made that changes to the surfaces of particles and fibres may reduce their toxicity and even pathogenicity. During this period a strong focus was placed on elucidating the mechanisms involved in particle and fibre toxicity and pathogenicity including inflammation, fibrosis, DNA damage and carcinogenesis.

With the advent of nanotechnology during the last decade, it was possible to show that there was much less new than was initially thought in Nanotoxicology. It was possible to use the physicochemical properties which were already identified for particles and fibres for defining NP toxicity. This could also be applicable to NPs with the important premise that NPs are separate entities with different chemical compositions capable of durable independent existence without disintegration over time to its chemical constituents. NP safety, health effects and exposure patterns also differ from their very chemical constituents. Hence, similar to larger sized particles and fibres, the assessment of their biodurability and subsequent long-term health effects should also be considered in addition to their short-term toxic effects.

### Risk management and governance for particles and fibres

**Fig. 7:**
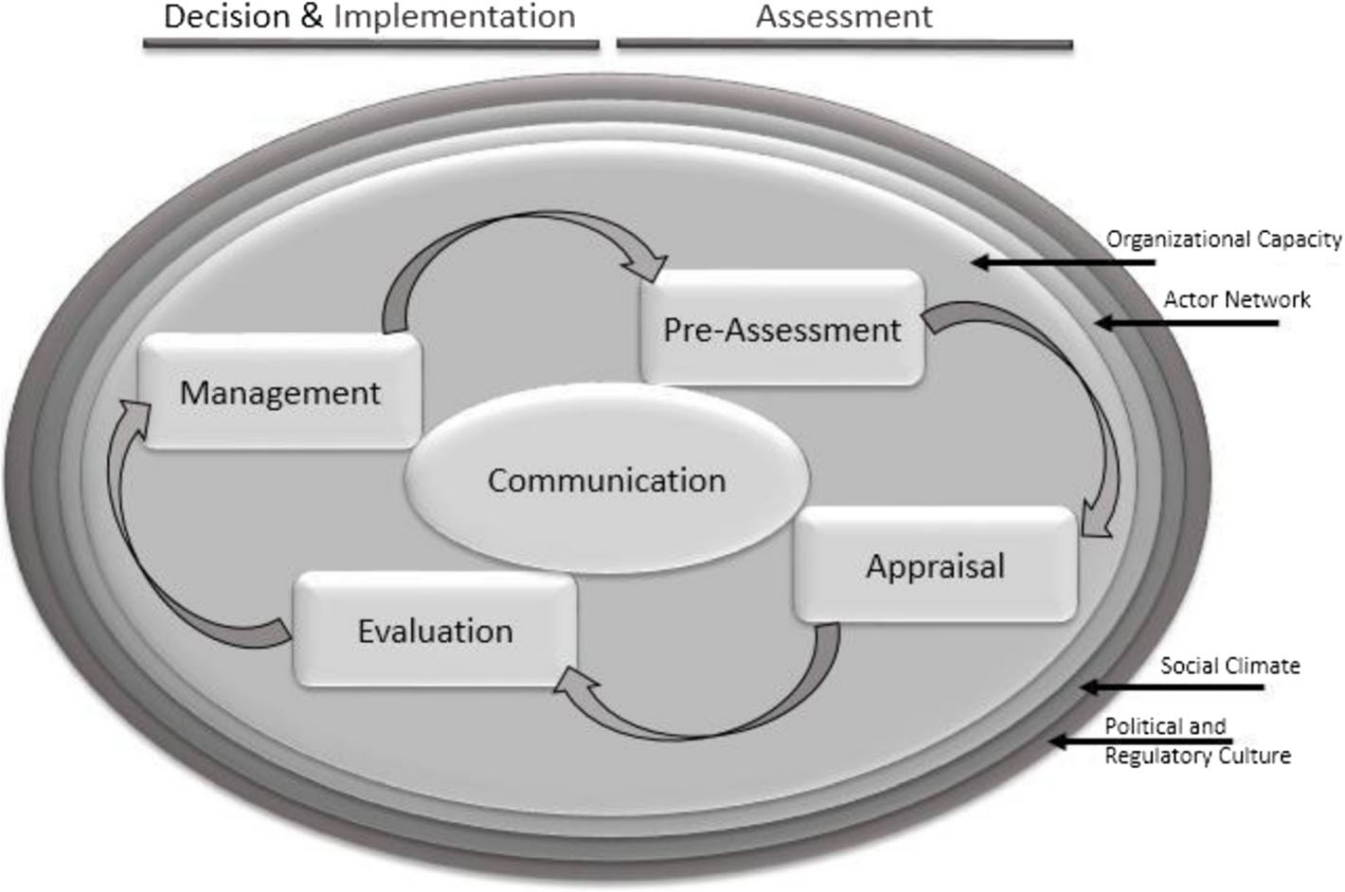
Example of a modern risk governance framework including a wide range of stakeholder communities (adapted by authors from IRGC, http://www.irgc.org/risk-governance/irgc-risk-governance-framework/, accessed July 17, 2015)

Risk governance serves as an organizational framework through which the critical elements of risk assessment, management, and communication are applied to the scientific and technological solutions for local, regional, and global issues. Through risk governance, the beneficial and adverse impacts of such solutions are understood, and using principles of cooperation and participation, effective policy decisions are made. Over the last 20 years, as the concept of risk governance has evolved, its scope has become more comprehensive, the number of stakeholder communities larger, and its practical application daunting Fig. 7 [416]. Also for nanomaterials (including NPs) risk governance, a series of risk governance strategies have been developed [417, 418]. In assessing scientific and technical products, a balance between innovation – the process that provides a new product or solution that is technologically possible and viable in the market place – and sustainability – the process that considers what is bearable and equitable for society, the environment, and the economy across the life cycle of the solution – must be struck. The challenges of stakeholder involvement in risk governance decision making are complicated by difficulty identifying and engaging the appropriate stakeholder communities, the limited coordination between government and non-government communities, and the inability of governments to agree on the principles and elements of risk governance. Perhaps the biggest challenge is the identification of a convening authority (or authorities) with the multidisciplinary expertise, neutrality, and objectivity needed to convene communities and governments within, and across, nations for consensus decisions. As one analyzes the complexity and challenges represented by this approach, one might indeed ask if the scope and organization of risk governance has made it too big to succeed and if a need has arisen to create a more focused, adaptive, and practical risk governance approach.

At the core of risk governance lays the continuous assessment, appraisal, evaluation and management of the risks. This process translates governance goals into concrete strategies for dealing with the risks posed by particles and fibres. Approaches to deal with hazardous dusts focused in the past on hazards and appropriate controls measures [419]. This has evolved to approaches that appraise the risk, which is the product of hazard and exposure, before defining strategies to manage this risk (e.g. [420]). To understand the risk of particles and fibres, it is necessary to collect basic information on the physico-chemical properties such as chemical composition, size, and shape, biopersistence, and reactivities; testing results such as acute and chronic toxicity; and dosing information such as bio-kinetics, exposure levels, frequencies and duration of exposure to consumers, workers and the general population. In a second step, these data are combined to appraise the risk in terms of people harmed, disabled or killed, and related metrics such as disability-adjusted life years or cost to victims, companies, insurance and society. Next, risk management measures are defined that aim to reduce the risk to acceptable levels. It is good occupational hygiene practice to further reduce risks that are easy to reduce. Considerable attention was given recently to nanomaterial and in particular NP risks and the knowledge gaps that science has to fill [5]. In addition to the challenges faced with controlling the risk from larger particles and fibres, NPs bring along two special challenges: first, materials often behaves differently at the nanoscale lending them different physico-chemical properties. Second, NPs were found to translocate (as discussed above) to different target organs compared to micron sized particles. Both of these challenges imply that data obtained in the past for larger particles of these materials may no longer be valid for the nanoform and a series of novel strategies has been advanced to identify properties that best predict NPs’ risk potential [421, 422]. Taken together, modern risk governance and risk management theories propose assessing risks in a comprehensive framework that aims at reducing the risk and the uncertainty related to it. In such a framework, one needs to understand the likely exposure levels and how they relate to release from products and production processes, how these concentration ranges translate into dose-response distribution functions and how they can be combined with dose-response functions and uncertainties of the NPs’ hazards. By focusing on optimizing the risk rather than addressing exposures and hazards individually, considerable gains are expected both in risk reduction as well as in terms of the time and cost required to reach acceptable risk levels.

## Conclusions

Albeit that in recent year substantial efforts have been undertaken to understand the hazard and health risk related to exposure to nanomaterials, much can still be learned from general particle toxicology. There are major similarities in terms of the effects that particles in wide size range can induce, in particular those that are referred to as being poorly soluble. Marked differences can be seen in deposition, translocation and clearance, in particular for inhalation exposures. From the more sophisticated tools that can now be applied, it has become evident that even poorly soluble particles can dissolve and sometime cause new particle formation, often seen in organs distant to the port of entry. Albeit that no specific new toxicological effects have been observed for nano-sized particles, the research of this group of particles have made people realize that also adverse effects can be localized in other organs than those that become initially in contact with particles.

## Abbreviations

5Bs: Bioavailability, Biopersistence, Bioprocessing, Biomodification, and Bioclearance (of nanoparticles)
ABB: Air-Blood-Barrier
AM: Alveolar Macrophages
AuNP: Gold NanoParticle
BAL: BronchoAlveolar Lavage
BALT: Bronchus-Associated Lymphoid Tissue
BaP: Benzo-a-Pyrene
BMD: BenchMark Dose
BSA: Bovine Serum Albumin
CNS: Central Nervous System
CPC: Condensation Particle Counter
EC: Elemental Carbon
EC1: Epithelial type 1 Cells
EC2: Epithelial type 2 Cells
EELS: Electron energy-loss spectroscopy
EPR: Enhanced permeability and retention
GIT: Gastro-intestinal-tract
HEC: Human Equivalent Concentration
HRTEM: High Resolution Transmission Electron Microscopy
ID: Initial Dose
IM: Interstitial Macrophages
IrNP: Iridium NanoParticle
IT: IntraTracheal
LOAEL: Lowest Observed Adverse Effect Levels
LT-MC: Long-Term Macrophage-mediated Clearance
MCC: MucoCiliary Clearance
MCNs: MiCellar Nanocomplexes
MPPD: Multiple Path Particle Dosimetry
N-N: Normal to Normal heart beat rate
NOAEC: No Observed Adverse Effect Concentration
NOAELs: No Observed Adverse Effect Levels
NOM: Natural Organic Matter
NP(s): NanoParticle(s)
OPC: Optical Particle Counter
PEGylation: Modification of (often medical) particle surfaces with PolyEthylene Glycol
PM: Particulate Matter
PM10: PM smaller than 10 μm
PM2.5: PM smaller than 2.5 μm
PSL: PolyStyrene Latex
RR50: the frequency of beat to beat heart intervals fluctuating by more than 50 msec
SEM: Scanning Electron Microscopy
SP-A: Surfactant Protein A
SP-D: Surfactant Protein D
sp-ICP-MS: single particle Induction Coupled Mass Spectrometry
STEM: Scanning Transmission Electron Microscopy
TEM: Transmission Electron Microscopy
TiO2-NP: Titanium dioxide NanoParticle
UFP: UltraFine Particles
UICC: Union for International Cancer Control
