# Correction to: Particle toxicology and health - where are we?

**DOI:** 10.1186/s12989-019-0308-2

**Published:** 2019-06-27

**Authors:** Michael Riediker, Daniele Zink, Wolfgang Kreyling, Günter Oberdörster, Alison Elder, Uschi Graham, Iseult Lynch, Albert Duschl, Gaku Ichihara, Sahoko Ichihara, Takahiro Kobayashi, Naomi Hisanaga, Masakazu Umezawa, Tsun-Jen Cheng, Richard Handy, Mary Gulumian, Sally Tinkle, Flemming Cassee

**Affiliations:** 1 Swiss Centre for Occupational and Environmental Health (SCOEH), Binzhofstrasse 87, CH-8404 Winterthur, Switzerland; 2 Institute of Bioengineering and Nanotechnology, Agency for Science, Technology and Research (A*STAR), Singapore, Singapore; 3 Institute of Epidemiology, Helmholtz Center Munich – German Research Center for Environmental Health, Neuherberg, Munich, Germany; 4 Department of Environmental Medicine, University of Rochester, Rochester, NY USA; 5 University of Kentucky, Lexington, KY USA; 6 School of Geography, Earth and Environmental Sciences, University of Birmingham, Birmingham, UK; 7 Department of Biosciences, Allergy Cancer BioNano Research Centre, University of Salzburg, Salzburg, Austria; 8 Tokyo University of Science, Tokyo, Japan; 9 Jichi Medical University, Simotsuke, Japan; 10 National Institute for Environmental Studies, Tsukuba-City, Japan; 11 Aichi Gakusen University, Okazaki, Japan; 12 National Taiwan University, Taipei, Taiwan; 13 School of Biological Sciences, Plymouth University, Plymouth, UK; 14 National Institute for Occupational Health and Haematology and Molecular Medicine, University of the Witwatersrand, Johannesburg, South Africa; 15 Science and Technology Policy Institute, Washington, DC, USA; 16 National Institute for Public Health and the Environment (RIVM), Bilthoven, The Netherlands; 17 Institute for Risk Assessment Studies (IRAS), Utrrecht University, Utrecht, The Netherlands

**Correction to: Part Fibre Toxicol**

**https://doi.org/10.1186/s12989-019-0302-8**

After the publication of this article [1] it was hihglighted that the number of deaths related to natural disasters was incorrectly reported in the second paragraph of the Hazards from Natural particulates and the evolution of the biosphere section. This correction article shows the correct and incorrect statement. This correction does not change the idea presented in the article that from an evolutionary view point, natural disasters account only for a small fraction of the people on the planet. The original article has been updated.

**Corrected statement:**

In comparison, today natural disasters account for less than 1 death per million inhabitants per year.

**Incorrect statement:**

In comparison, today natural disasters account for some 375 million deaths per year.
